# Structural Brain Correlates of Childhood Inhibited Temperament: An ENIGMA-Anxiety Mega-analysis

**DOI:** 10.1016/j.jaac.2022.04.023

**Published:** 2022-06-13

**Authors:** Janna Marie Bas-Hoogendam, Rachel Bernstein, Brenda E. Benson, Kristin A. Buss, Kelley E. Gunther, Koraly Pérez-Edgar, Giovanni A. Salum, Andrea P. Jackowski, Rodrigo A. Bressan, André Zugman, Kathryn A. Degnan, Courtney A. Filippi, Nathan A. Fox, Heather A. Henderson, Alva Tang, Selin Zeytinoglu, Anita Harrewijn, Manon H.J. Hillegers, Tonya White, Marinus H. van IJzendoorn, Carl E. Schwartz, Julia M. Felicione, Kathryn A. DeYoung, Alexander J. Shackman, Jason F. Smith, Rachael M. Tillman, Yvonne H.M. van den Berg, Antonius H.N. Cillessen, Karin Roelofs, Anna Tyborowska, Shirley Y. Hill, Marco Battaglia, Marco Tettamanti, Lea R. Dougherty, Jingwen Jin, Daniel N. Klein, Hoi-Chung Leung, Suzanne N. Avery, Jennifer Urbano Blackford, Jacqueline A. Clauss, Elizabeth P. Hayden, Pan Liu, Matthew R.J. Vandermeer, H. Hill Goldsmith, Elizabeth M. Planalp, Thomas E. Nichols, Paul M. Thompson, P. Michiel Westenberg, Nic J.A. van der Wee, Nynke A. Groenewold, Dan J. Stein, Anderson M. Winkler, Daniel S. Pine

**Affiliations:** Drs. Bas-Hoogendam and Westenberg are with Leiden University, Leiden, the Netherlands.; Drs. Bas-Hoogendam and van der Wee are with Leiden University Medical Center, Leiden, the Netherlands.; Drs. Bas-Hoogendam, Westenberg, and van der Wee are with Leiden Institute for Brain and Cognition, Leiden, the Netherlands.; Drs. Bas-Hoogendam, Benson, Zugman, Filippi, Winkler, and Pine, and Ms. Bernstein are with the National Institute of Mental Health, Bethesda, Maryland.; Drs. Buss and Pérez-Edgar and Ms. Gunther are with Pennsylvania State University, University Park.; Dr. Salum is with Hospital de Clínicas de Porto Alegre, Universidade Federal do Rio Grande do Sul, Porto Alegre, Brazil.; Drs. Jackowski and Bressan are with Federal University of São Paulo, São Paulo, Brazil.; Dr. Degnan is with The Catholic University of America, Washington, DC.; Drs. Filippi, Fox, Tang, Zeytinoglu, Shackman, Smith, Tillman, and Dougherty and Ms. DeYoung are with the University of Maryland, College Park.; Dr. Henderson is with the University of Waterloo, Waterloo, Ontario, Canada.; Dr. Harrewijn is with Erasmus University Rotterdam, Rotterdam, the Netherlands.; Drs. Hillegers and White are with Erasmus University Medical Center, Rotterdam, the Netherlands.; Dr. van IJzendoorn is with University College London, United Kingdom.; Drs. Schwartz and Clauss are with Massachusetts General Hospital, Harvard Medical School, Boston.; Ms. Felicione is with Tufts University, Medford, Massachusetts.; Drs. van den Berg, Cillessen, Roelofs, and Tyborowska are with Radboud University Nijmegen, the Netherlands.; Dr. Hill is with the University of Pittsburgh School of Medicine, Pennsylvania.; Dr. Battaglia is with the University of Toronto, Ontario, Canada, and the Centre for Addiction and Mental Health, Toronto, Ontario, Canada.; Dr. Tettamanti is with the University of Trento, Rovereto, Italy.; Dr. Jin is with the University of Hong Kong, Hong Kong.; Drs. Klein and Leung are with Stony Brook University, New York.; Drs. Avery and Blackford are with Vanderbilt University Medical Center, Nashville, Tennessee.; Dr. Blackford is also with the University of Nebraska Medical Center, Omaha.; Drs. Hayden, Liu, and Vandermeer are with Western University, London, Ontario, Canada.; Dr. Liu is also with North Dakota State University, Fargo.; Drs. Goldsmith and Planalp are with the University of Wisconsin–Madison.; Dr. Nichols is with the University of Oxford, United Kingdom.; Dr. Thompson is with the University of Southern California, Marina del Rey.; Drs. Groenewold and Stein are with the University of Cape Town, Cape Town, South Africa.

## STUDY SYNOPSIS

### Introduction Summary

Temperament involves stable behavioral and emotional tendencies that differ between individuals, which can be first observed in infancy or early childhood and relate to behavior in many contexts and over many years.^1^ One of the most rigorously characterized temperament classifications relates to the tendency of individuals to avoid the unfamiliar and to withdraw from unfamiliar people, objects, and unexpected events. This temperament is referred to as behavioral inhibition or inhibited temperament (IT).^2^ IT is a moderately heritable trait^1^ that can be measured in multiple species.^3^ In humans, levels of IT can be quantified from the first year of life through direct behavioral observations or reports by caregivers or teachers. Similar approaches as well as self-report questionnaires on current and/or retrospective levels of IT^1^ can be used later in life.

Variations in IT are present on a continuous scale within the population, and research suggests that about 20% of young children are characterized by high IT,^4^ which is in general stable over time.^5^ Considerable data suggest that this high childhood IT (cIT) has adverse long-term consequences: infants with cIT often become more reserved adults, and, on average, such infants exhibit poorer outcomes than noninhibited infants with respect to social relationships and internalizing psychopathology.^6^ More specifically, almost half of all children with elevated and stable cIT will develop social anxiety disorder later in life compared with only 12% of noninhibited children.^7^ Thus, cIT predicts risk for later psychopathology, especially social anxiety disorder.^8,9^

Several neuroimaging studies have examined neurobiological correlates of cIT. Such research is important, as brain characteristics—including brain structure, function, and connectivity—may mediate the cIT-related risk for poor outcomes.^10^ Previous studies have linked cIT to the structure and function of brain networks involved in emotion perception, experience, and regulation.^1^ These brain networks involve the dorsal (caudal) and ventral (rostral) anterior cingulate cortex, insula, amygdala, dorsolateral and medial prefrontal cortex, orbitofrontal cortex, and striatum (cf.^1,10^), all of which have also been implicated in the familial risk for social anxiety disorder.^11^ In addition, translational work on anxious temperament has indicated involvement of the hippocampus.^3,12^ Despite this progress, the few available studies on the neural structural correlates of cIT are often restricted to specific regions of interest, while, to the best of our knowledge, cortical surface area and cortical thickness have been examined in only one study with an exploratory approach.^13^ Furthermore, most findings with respect to brain structure are unique to a specific sample, and crossstudy comparisons are limited by relatively small sample sizes and failure to consider potential modifying variables such as age and biological sex.

In this ENIGMA-Anxiety project,^14^ we aim to extend previous work by examining brain structure associated with cIT in a large dataset, assembling data acquired at 12 research centers worldwide (17 samples, *N* = 4,681) (Table 1). Compared with the individual studies, this new study is better powered owing to the larger number of research participants available for analysis. Moreover, by combining data through a mega-analytic approach, the present study facilitates the differentiation of consistent, generalizable findings from false-positive findings that could emerge from studies with smaller samples. Such work has the potential to establish reproducible anatomical correlates and could inform the development of mechanistic studies and intervention research with clinical relevance.^15^

We expect to corroborate findings in brain circuits found previously (involved in processing fear, reward, and emotion regulation),^1,10^ with small-to-medium effect sizes. We hypothesize that structural alterations in brain regions involved in these processes, in particular gray matter volumes of multiple subcortical structures (amygdala, hippocampus, striatum including caudate and putamen), and characteristics of several frontal and temporal cortical areas (orbitofrontal cortex, anterior cingulate cortex, insula superior temporal gyrus, transverse gyrus, fusiform gyrus) are neural substrates of cIT.

### Method Summary

This ENIGMA-Anxiety Working Group project^14^ will include individual participant data assembled from studies in which participants underwent magnetic resonance imaging scanning (T1-weighted anatomical magnetic resonance imaging scans) between 6 and 25 years of age. Regardless of age at the time of scanning, all participants will be phenotyped for cIT (defined as age ≤12 years). These temperament assessments could be behavioral observations in childhood, parental reports, or self-report questionnaires on current or retrospective temperament. We will perform a mega-analysis with a whole-brain approach (regional and vertex-wise; familywise error rate–corrected)^16^ and investigate the relation between cIT (continuous) and 3 distinct neuroanatomical metrics (determined using FreeSurfer software [https://surfer.nmr.mgh.harvard.edu/]), namely, volumes of subcortical structures, cortical thickness, and cortical surface area. Additionally, analyses will be performed in 3 subsets, based on the method and thus age at which cIT was determined: first (early-life) behavioral observations, second parental/teacher reports during childhood, and third self-report measures acquired during late childhood/adolescence. A fourth sensitivity analysis will exclude samples with retrospective measures of cIT.

### Significance Summary

This initiative is the first mega-analysis of brain structure associated with the temperamental risk for developing internalizing psychopathology. This provides the possibility of detecting novel cIT-related brain alterations and clarifying inconsistent findings of prior work.^10^ Mega-analyses combine existing datasets to increase the overall sample size. This is particularly valuable for data acquired in vulnerable participants, who are often difficult to recruit. Such studies exemplify next-generation science: previous studies within the ENIGMA Consortium have resulted in important insights in the neurobiology of psychiatric conditions.^17^ These discoveries reflect the advantages of large-scale data analyses for testing the reproducibility and robustness of neuroimaging findings.^17^ We expect the current project to provide similar insights, increasing our understanding of the development of psychopathology in youth at risk. In addition, by preregistering the study in advance of performing the analyses, we hope to contribute to a reduction of the potential publication bias in the field and to advance a more complete scientific record on this topic (cf.^18^).

## Supplementary Material

Supplement

## Figures and Tables

**TABLE 1:** Dataset for the ENIGMA-Anxiety Mega-Analysis on Childhood Inhibited Temperament

Sample (location)	Type of sample	*N* (*n* female) with MRI and cIT data		Design^a^	Age at MRI scan, range (mean ± SD)		Age at cIT phenotype, range (mean ± SD)		Measure of cIT
Brains study (Pennsylvania State University, State College, Pennsylvania)	Oversampled for high/low cIT	130	(72)	C	9.2–13.2 y	(10.8 ± 1.0)	9.2–13.2 y	(10.8 ± 1.0)	BIQ–parent rated
Brazilian High Risk Cohort (National Institute of Developmental Psychiatry for Children and Adolescents [INPD], São Paulo, Brazil)	Community sample and high-risk sample of children with increased familial risk for mental disorders	678	(290)	C	5.8–13.0 y	(9.7 ± 1.6)	5.8–13.0 y	(9.7 ± 1.6)	EATQ-R–shyness scale
Cohort 3/4 (University of Maryland, College Park, Maryland)	Community sample: prospective longitudinal study of infants thought likely to display behavioral inhibition later in infancy and early childhood	95	(51)	L	13.3–21.1 y	(18.0 ± 1.9)	Around 24 mo	(no data at individual level)	Standard laboratory observations: composite score of stranger, robot, tunnel episodes
Generation R, sample with behavioral observations (Erasmus University Medical Center, Rotterdam, the Netherlands)	Longitudinal community sample	584	(297)	L	8.7–12.0 y	(10.2 ± 0.6)	34.7–44.2 mo	(37.4 ± 1.4)	Standard laboratory observations: stranger approach and jumping spider episode from Lab-TAB
Generation R, sample with questionnaire data (Erasmus University Medical Center, Rotterdam, the Netherlands)	Longitudinal community sample	1,982	(1,030)	L	8.6–12.0 y	(10.0 ± 0.5)	4.5–11.8 mo	(6.7 ± 1.1)	IBQ-r–fear subscale
Maryland-PAX (University of Maryland, College Park, Maryland)	30-mo longitudinal study of a sample of first-year university students enriched for internalizing risk	220	(109)	C	18–19 y	(18.3 ± 0.4)	Retrospective: remembered inhibited behaviors in childhood		RMBI
Maryland-TAX (University of Maryland, College Park, Maryland)	Cross-sectional community sample	53	(28)	C	13–17 y	(15.0 ± 1.2)	Retrospective: remembered inhibited behaviors in childhood		RSRI–child rated
Nijmegen Longitudinal Study (Radboud University, Nijmegen, the Netherlands)	Longitudinal community sample	71	(31)	L	17 y		1.20–1.28 y	(1.24 ± 0.02)	Standard laboratory observations at age 15 mo: stranger and robot episodes
Pittsburgh (University of Pittsburgh School of Medicine, Pittsburgh, Pennsylvania)	High- and low-risk (control) children/adolescents from ongoing family studies	15	(3)	L	19.2–24.8 y	(21.5 ± 1.7)	4.1–6.4 y	(5.1 ± 0.7)	Laboratory observations during peer play
San Raffaele (Vita-Salute San Raffaele University and San Raffaele Scientific Institute, Milan, Italy)	Community sample	20	(8)	L	13–16 y	(14.8 ± 1.1)	8–10 y	(9.1 ± 0.7)	Empirical composite index
SDAN (NIMH, Bethesda, Maryland)	Treatment-seeking children and control group of healthy volunteers	55	(26)	C	7.3–14.6 y	(10.3 ± 1.7)	8.0–12.8 y	(10.4 ± 1.5)	BIQ–child rated
Stony Brook Temperament Study (Stony Brook University, Stony Brook, New York)	Community sample; MRI subsample oversampled for youth with temperamental high negative emotionality, low positive emotionality, and high behavioral inhibition at age 3	74	(31)	L	9–12 y	(10.2 ± 0.9)	2.9–4.0 y	(3.4 ± 0.3)	Lab-TAB: 3 Kagan-like tasks around age 3
TOTS (University of Maryland, College Park, Maryland)	Longitudinally followed sample of children selected at age 4 mo based on their behavior in the laboratory	96	(56)	L	9.1–19.5 y	(11.4 ± 2.1)	1.9–2.7 y	(2.1 ± 0.2)	Standard laboratory observations (composite score of stranger, robot, tunnel episodes)
Vanderbilt–children (Vanderbilt University Medical Center, Nashville, Tennessee)	Study with extreme discordant phenotypes approach: inhibited and uninhibited children at the extreme ends	55	(33)	C	8–12 y	(9.3 ± 1.1)	8–12 y	(9.3 ± 1.1)	BIQ–child rated
Vanderbilt–young adults (Vanderbilt University Medical Center, Nashville, Tennessee)	Study with extreme discordant phenotypes approach: inhibited and uninhibited young adults at the extreme ends	150	(83)	C	18–25 y	(21.8 ± 2.0)	Retrospective: remembered inhibited behaviors in childhood		RSRI
Western University (The Brain and Mind Institute, Western University, London, Ontario, Canada)	Children selected based on presence/absence maternal depression	87	(38)	L	9.2–12.4 y	(11.1 ± 0.7)	3.0–4.0 y	(3.4 ± 0.3)	Lab-TAB: risk room, stranger approach, and jumping spider
Wisconsin Twin Project–RDoC twin study (University of Wisconsin–Madison, Madison, Wisconsin)	Longitudinally followed samples of twins, recruited from statewide birth records for birth cohorts 1989–2004	316	(145)	L	15.1–23.9 y	(17.5 ± 1.6)	6.5–9.0 y	(7.5 ± 0.5)	Ratings on approach and shyness from 3-h home visit and scores from videotaped reactions to “Conversation With a Stranger” episode of Lab-TAB
Total *N*		4,681	(2,331)						

***Note***: BIQ = Behavioral Inhibition Questionnaire; cIT = childhood inhibited temperament; EATQ-R = Revised Early Adolescent Temperament Questionnaire; IBQ-r = Infant Behavior Questionnaire - revised; Lab-TAB = Laboratory Temperament Assessment Battery; MRI = magnetic resonance imaging; NIMH = National Institute of Mental Health; PAX = prospective anxiety; RDoC = Research Domain Criteria; RMBI = Retrospective Measure of Behavioural Inhibition; RSRI = Retrospective Self-Report of Inhibition; SDAN = Section on Development and Affective Neuroscience; TAX = teen anxiety; TOTS = Temperament Over Time Study.

a With respect to time point temperament assessment and MRI scan for data used in this study: C = cross-sectional; L = longitudinal.
