# Supplementary material for: Structural Brain Correlates of Childhood Inhibited Temperament: An ENIGMA-Anxiety Mega-analysis
Source: J Am Acad Child Adolesc Psychiatry. Author manuscript; Available in PMC 2022 Sep 1. (PMC9434711; doi:10.1016/j.jaac.2022.04.023)
Supplement: Supplement [file NIHMS1819229-supplement-Supplement.docx]

**Registered Report**

**Structural brain correlates of childhood inhibited temperament: an ENIGMA-Anxiety mega-analysis**

**Supplemental Materials Stage 1 Manuscript**

***Bas-Hoogendam et al.***

**Supplemental Methods**

**Questionnaires on psychopathology**

All research sites were asked to provide as much questionnaire data on psychopathology as possible – cf. the methods described in Bas-Hoogendam et al.^1^. This concerned the following questionnaires with respect to anxiety disorders: the Hamilton Anxiety Rating Scale ^2^, Penn State Worry Questionnaire ^3^, Generalized Anxiety Disorder 7-Item questionnaire ^4^, State Trait Anxiety Inventory ^5^, Anxiety Sensitivity Index ^6^, Beck Anxiety Inventory ^7^, Liebowitz Social Anxiety Scale ^8^, Panic and Agoraphobia Scale ^9^, Agoraphobic Cognition Questionnaire ^10^, Panic Disorder Severity Scale ^11^ and the Screen Child Anxiety Related Disorders ^12^.

Furthermore, we asked for data on the Beck Depression Inventory II ^13^, the Children’s Depression Inventory ^14^, and Hamilton Depression Rating Scale ^15^.

Not all research sites had available data on these variables, therefore, these data were not included in the analyses. Descriptive information and scores on these questionnaires for each sample are provided in **Supplemental Table 3.**

**Information for each sample: diagnostic interviews, clinical questionnaires and assessment of temperament**

[Brains sample 4](#_Toc91167178)

[Brazilian High Risk Cohort (BHRC) 6](#_Toc91167179)

[Cohort 3/4 8](#_Toc91167180)

[Generation R 10](#_Toc91167181)

[Maryland – PAX sample 13](#_Toc91167182)

[Maryland – TAX sample 15](#_Toc91167183)

[Nijmegen Longitudinal Study on Child and Infant Development 17](#_Toc91167184)

[Pittsburgh 22](#_Toc91167185)

[San Raffaele 25](#_Toc91167186)

[SDAN 29](#_Toc91167187)

[Stony Brook Temperament Study 30](#_Toc91167188)

[TOTS 34](#_Toc91167189)

[Vanderbilt - children 37](#_Toc91167190)

[Vanderbilt – young adults 39](#_Toc91167191)

[Western University 41](#_Toc91167192)

[Wisconsin Twin Project 43](#_Toc91167193)

# Brains sample

**Publications**: ^16–25^

**In- and exclusion criteria** ^17^

**﻿**The sample consisted of 9–12-year-olds, of families which were recruited through a university database of families interested in participating in research studies, community outreach, and word-of-mouth. The study was part of a larger study on temperament, attention, and anxiety. Participants were screened using parental report on the Behavioral Inhibition Questionnaire (BIQ) ^26^. Children who met BI cut-off scores (>120 in BIQ Total score or >60 in BIQ Social novelty; ~25% of children screened) were identified and oversampled, while children below cut-off were recruited as a gender- and age-matched non-BI comparison group. Cut-off scores were based on previous studies of extreme temperament in children ^27^. Exclusionary criteria included severe psychiatric diagnosis (e.g. bipolar disorder), IQ below 70, or severe medical illness. Parents and children provided written consent/assent and the Institutional Review Board approved this study.

**Diagnostic interview** ^16,17^

Social anxiety symptoms and major depressive disorder were assessed via parent-report on the computerized Diagnostic Interview Schedule for Children version 4 (C-DISC 4; ^28^). A trained research assistant conducted the semi-structured interview, in which parents judged DSM-IV symptoms as either present (‘yes’) or absent (‘no’).

Anxiety symptoms were measured using the parent-report version the Screen for Child Anxiety Related Emotional Disorders (SCARED) ^12^, a 41-item instrument assessing symptoms of panic disorder, generalized anxiety, separation anxiety, social phobia, and school phobia defined in the Diagnostic and Statistical Manual of Mental Disorders (DSM-IV). Parents rated the frequency with which their children experience each symptom on three-point scales (0 = “almost never”, 1 = “sometimes”, and 2 = “often”). Sub-scale scores were summed to create the total score. The SCARED has satisfactory psychometric properties in both clinical ^12^ and community samples^29^ and it offers a valuable tool to predict specific anxiety disorders in clinically-referred youths ^30^. It had good internal consistency in the present study (Cronbach’s alpha = .90).

**Measures of temperament**^16^

Behavioral inhibition (BI) was assessed using the Behavioral Inhibition Questionnaire (BIQ) ^26^, a 30-item instrument that measures the frequency of BI-linked behavior in the domains of social and situational novelty (plus a summed total score) on a seven-point scale ranging from 1(“hardly ever”) to 7 (“almost always”). Four questions were edited to be more appropriate for the target age range in the current study (e.g., reference to preschool, kindergarten, and childcare was removed for the question: “Happily separates from parent(s) when left in new situations for the first time (e.g., kindergarten, preschool, childcare)”). The questionnaire has adequate internal consistency, construct validity, and validity in differentiating behaviorally inhibited from non-inhibited children ^26^, parent reports on the BIQ correlate with laboratory observations of BI in social contexts ^31^, and the BIQ had good internal consistency in the present study (Cronbach’s alpha = .91).

In the present study, we will use the total score on the BIQ as indicator of cIT.

# Brazilian High Risk Cohort (BHRC)

**Publications:** ^32,33^

**In- and exclusion criteria** ^32,34,35^

As summarized in Axelrud and colleagues ^34^, “the screening stage for the BHRC took place in 2009/2010 in public schools from two cities in Brazil (Porto Alegre and São Paulo), including a total of 9937 children. Eligibility criteria were being 6–12 years old at enrolment and being registered by a biological parent who could provide information about the children’s behavior. From the screening sample, 2511 children were selected and evaluated at baseline in 2010/2011. Among this subsample, 1554 children were at risk of mental illness, established using family history and the current presence of symptoms, and 957 were randomly selected. A subset of 741 participants underwent MRI scans and 726 of these also underwent psycho-pathology assessment. Parents of the participants and participants provided written or verbal consent. The Ethics Committee of the University of São Paulo approved the study.”

For the present study, we selected participants aged < 13 years at baseline, with imaging data and EATQ-R data.

**Diagnostic interview**

As described in Hoffmann et al.^35^, “mental disorders were assessed using the Brazilian Portuguese version ^36^ of the DAWBA ^37^. This structured interview was administered to biological parents by trained lay interviewers. Responses, as well as structured answers, were then evaluated by a total of nine certified child psychiatrists, which confirmed, refuted or altered the initial computerized diagnosis. All of them were trained and supervised jointly by a senior child psychiatrist with extensive experience in rating the DAWBA. To perform reliability analysis of the rating procedure, a sub-sample of 200 subjects received a second rating by a trained child psychiatrist. We selected subjects divided equally into DAWBA bands ^37^. DAWBA bands represent computer-generated categories based on answers to the DAWBA questions that provide information to the rater concerning the probability of a positive diagnosis (< 0.1%, ~ 3%, ~ 15%, ~ 50% and higher than 70%). The second rater was informed that the 200 cases (40 cases from each band) did not represent the population distribution of mental disorders. Inter-rater agreement was above 90% for all diagnosis and kappa values ranging from 0.72 for hyperkinetic disorders and 0.84 for emotional disorders ^38^. Diagnoses are related to diagnostic criteria from the Diagnostic and Statistical Manual of Mental Disorders, 4th edition.”

**Measure of temperament** ^35^

Temperament was assessed with the Brazilian-Portuguese self-report version of the early adolescent temperament questionnaire (EATQ-R)^38,39^. This questionnaire is a 65-items Likert scale, ranging from 1 (always false) to 5 (always true), containing 12 subscales (4–7 items each). Five temperament factors were used, namely effortful control, fear, frustration, shyness and surgency ^40,41^.

In the present study, we will use the sum score of the shyness items of the EATQ-R as an index of cIT.

# Cohort 3/4

**Publications:** ^42–44^

**In- and exclusion criteria**

As described by Shechner et al.^43^, “participants were a subsample of individuals who were selected at 4 months of age and assessed for BI at ages 14 months and 24 months, and for social reticence at 4 and 7 years of age – cf. Fox and colleagues ^42^. At each time point, parental ratings of shyness were also collected. Individuals taking psychotropic medications, reporting acute psychopathology in need of immediate treatment, taking recreational drugs, or having any contraindications to MRI (e.g., permanent retainer) were excluded from the current study. All other individuals from the longitudinal study were asked to participate if they were physically healthy based on medical examination and history and had an IQ of > 70.”

**Diagnostic interview**

The presence of current or lifetime psychiatric disorder was assessed by the Structured Clinical Interview for DSM IV (SCID) ^45^. Anxiety levels were assessed in two ways: (1) the Beck Anxiety Inventory (BAI) ^7^ was used as a measure of trait anxiety, and (2) the State subscale of the State Trait Anxiety Inventory (STAI-S) ^5^ was used as a measure of state anxiety at the time of the scan.

**Measures of temperament**

Inhibited behavior to novel stimuli was coded at 14 and 24 months ^42^. Behavioral scores were standardized at each time point. For the present study, we will use the ‘BI-classic-24’ index, composed of scores on the stranger, robot and tunnel episodes of the Laboratory Temperament Assessment Battery – preschool version ^46^. Mothers also reported their child’s social fear at 14 and 24 months using the Toddler Behavior Assessment Questionnaire ^47^.

# Generation R

**Publications:** ^48–52^

**Study design; in- and exclusion criteria**

For this project, we will use the data from the second neuroimaging wave of Generation R (children age 9 – 11) ^50^, because this imaging wave consisted of more children and was more representative of the overall Generation R sample when compared to the participants in the first imaging wave ^49^. As described in White et al (2018) ^50^, “﻿the children who were recruited were participants of the Generation R Study, which is a population-based longitudinal cohort study of child health and development based in ﻿Rotterdam, the Netherlands. An overview of the Generation R study design and population has been described in detail in ^53^. In brief, all pregnant women who were living within a well-defined region in Rotterdam (defined by postal codes) with a delivery data between April 2002 and January 2006 were invited to participate. A total of 9,778 mothers provided informed consent and were recruited. Rotterdam is ethnically diverse, with approximately 44% of the population being non-Dutch. Recruitment into Generation R reflects this diversity. Of the 9,778 mothers, 58% were Dutch, 9% Surinamese, 9% Turkish, 7% Moroccan, 3% Dutch Antillean, and 3% of Cape Verdian descent ^53^. Additional detailed measurements of fetal and postnatal growth and development have been conducted in a randomly selected subgroup of Dutch children (*n* = 1,232; known as the ‘Focus Cohort’) and their parents at 32 weeks gestational age and at the postnatal ages of 1.5, 6, 14, 24, 36 and 48 months. These additional evaluations on this subgroup were conducted in a Generation R dedicated research center. From the age of 5 years onwards, all willing children and their parents with the Generation R Study have had regular visits to a dedicated research center that includes advanced imaging facilities. The second wave of neuroimaging started in March 2013 with a total of 4,245 children visiting the MRI Centre and 4,087 children received a brain MRI scan, of which 3,992 fulfilled the Dutch laws of parental consent for research and of these 3,959 children completed a complete T1-weighted sequence.”

**Diagnostic interview**

**﻿**As outlined by White and colleagues ^50^, the Diagnostic Interview Schedule for Children-young child version (DISC-YC) was administered in subsample of the Generation R Study that was enriched for psychopathology ^54^, and this interview took place between the ages of 5- 8 years. The DISC-YC is a highly structured DSM-IV-based interview administered to caregivers of children aged 3–8 years. Six trained interviewers (including bilingual interviewers) administered the computer-assisted DISC-YC that determines the presence of disorders for a timeframe of 3 months, or 1-year for dysthymia and conduct disorder, by applying algorithms provided by the developer.

**Measures of temperament**

***Age 6 months (description from Jansen and colleagues, 2009*** ^52^***)*** “At the age of six months, infant temperament was assessed using an adapted version of the infant behavior questionnaire-revised (IBQ-R) ^55^. A detailed description of the changes has previously been described ^56^. Briefly, we assessed six scales of the IBQ-R: Activity Level (e.g. movements of arms and legs); Distress to Limitations (e.g. fussing or crying while in caretaking activities); Duration of Orienting (e.g. attention to a single object for extended periods of time); Sadness (e.g. general low mood); Fear (e.g. startle or distress to novelty or sudden changes in stimulation); and Recovery from Distress (e.g. rate of recovery from general arousal; ease of falling asleep). Internal consistencies for the adapted IBQ-R ranged from 0.70 for Duration of Orienting to 0.85 for Fear, which is comparable to the internal consistencies of the original IBQ-R ^55^.”

***Age 36 months*** ^51^ A subsample of the Generation R sample (‘Focus Cohort’; all of Dutch origin) visited the lab where stranger fear and the response to a jumping spider were assessed using the Laboratory Temperament Assessment Battery (Lab-TAB) ^57^.

In the present mega-analysis, we will use the scores on the Lab-TAB as index of cIT (sample with behavioral observations). For participants without these scores, we will use the IBQ- scores on the Fear subscale (sample with questionnaire data).

# Maryland – PAX sample

**Publications:** ^58–60^

**In- and exclusion criteria**

This prospective-longitudinal study focused on the emergence of anxiety disorders and depression and is described in more detail in Hur et al. (2020) ^58^ and in press. Participants were

first-year university students recruited from the University of Maryland. All subjects had normal or corrected-to-normal color vision; and reported the absence of lifetime neurologic symptoms, pervasive developmental disorder, very premature birth, medical conditions that would contraindicate MRI, and prior experience with noxious electrical stimulation. All subjects were free from a lifetime history of psychotic and bipolar disorders; a current diagnosis of a mood, anxiety, or trauma disorder (past 2 months), excepting subclinical (‘other specified’) diagnoses; severe substance abuse; active suicidality; and ongoing psychiatric treatment as determined by an experienced masters-level diagnostician using the Structured Clinical Interview for DSM-5 ^61^.

**Diagnostic interview:** Structured Clinical Interview for DSM-5 (SCID-5-RV) ^61^.

**Measures of temperament**

Participants completed the Adult Measure of Behavioral Inhibition (AMBI) and the Retrospective Measure of Behavioral Inhibition (RMBI) ^62^. The AMBI is a 16-item clinical research instrument developed in order to measure subjective reports of contemporaneous ‘trait’ inhibition. This instrument provides a dimensional quantitative measurement of the temperamental tendency to respond to social novelty and risk stimuli, with inhibition and avoidance. The Retrospective Measure of Behavioral Inhibition (RMBI) is an 18-item instrument for the retrospective adult reporting of shyness, reticence, and behavioral inhibition during childhood and early adolescence. This instrument was designed to capture the principal behavioral indices of “behavioral inhibition to the unfamiliar” as measured and observed in children when assessed in play-laboratory settings. This instrument is also a dimensional/quantitative measure. Higher scores on both measures indicate a greater degree of inhibition. Within the PAX-sample, scores on the AMBI and RMBI (total scores) were significantly correlated (*r*  = 0.47, *p* < 0.001).

For the current mega-analysis, we will use the total score for the RMBI as the cIT index.

# Maryland – TAX sample

**Publications**

*Not yet available.*

**In- and exclusion criteria**

Eighty-four participants between the ages of 13 and 17 years and their caregivers were recruited from advertisements distributed online (i.e., Facebook, listservs), flyers posted at community mental health clinics and broader community settings (i.e., coffee shops, local community centers), and referrals from other university research studies recruiting adolescents. Advertisements were designed to differentially target adolescents with high social anxiety using language inviting “shy” or “socially anxious” adolescents to participate in a study about brain function. Advertisements designed for adolescents without high levels of social anxiety used general language to invite participants to enroll, such as “Are you a teen?” or “Have a teen aged 13-17?”, and these advertisements were only distributed in general community settings.

To ensure inclusion of a clinically enriched sample that comprised both adolescents with social anxiety disorder and adolescents with low levels of social anxiety, participants completed a preliminary screening questionnaire online. The preliminary screening included a measure of the frequency of social anxiety disorder symptoms (the abbreviated Social Phobia and Anxiety Scale for Children; SPAIC-11 ^63^) and three additional questions designed to assess interference and distress from social anxiety symptoms using a 5-point Likert scale (*1 = Not at all, 5 = Extremely*). Individuals were invited to enroll if they met *any* of the following preliminary inclusion criteria: 1) obtaining a score of 16 or above on the SPAIC-11^63^; 2) indicating social anxiety interference or distress on the online screener prior to enrollment; and 3) obtaining a score of 6 or below on the abbreviated SPAIC-11 ^63^ *and* indicating low social anxiety interference and distress on the online screener prior to enrollment.

Participants in both groups were right-handed native English speakers with no history of head injury, neurological disorders, psychosis disorders, pervasive developmental disorders (e.g., autism) and bipolar disorder. Participants were free from MRI contraindications and were not currently using any psychotropic medications.

**Diagnostic interview**

The Mini-International Neuropsychiatric Interview For Children And Adolescents (MINI-KID ^64^) based on DSM-IV and ICD-10 criteria.

**Measures of temperament**

Within the TAX sample, five measures of inhibited temperament were acquired. Three were self-reports: the Current Self-Report of Inhibition (CSRI), the Retrospective Self-report of Inhibition (RSRI; focused on elementary and early middle school) ^65^ and the BIQ-A ^26^. In addition, a caregiver completed the CSRI and RSRI for their child. All five measures were highly correlated (all within-subject correlations *p* < 0.001, r > 0.55).

For the current mega-analysis, we will use the total score for the adolescent-reported RSRI as the cIT index.

# Nijmegen Longitudinal Study on Child and Infant Development

**Publications** ^66–68^

**In- and exclusion criteria at age 15 months**

(As described in Van Bakel et al.^68^ ): “The sample consisted of 129 physically healthy 15-month-old infants (67 boys, 62 girls) and their primary caregivers. Because earlier research has recommended that studies of the possible determinants of parenting be conducted in heterogeneous samples ^69^, the aim was to recruit such a sample in the present study. The recruitment of the families was based on the records from local health-care centers in the city of Nijmegen in The Netherlands. During 9 consecutive months, all families with a15-month-old baby (i.e., 639 families) living in districts with many young families from various socio-economic backgrounds were contacted. They were sent a recruitment letter explaining the goals of the study and were asked to return a card if interested in participating. Of the 174 families who replied, 129 parent–child dyads (the maximum attainable given the time and resources available for the project) were randomly selected for the study. The sample included

123 two-parent families and 6 single-parent families. In 3 families, the father was the primary caregiver of the child. In these cases, the mothers were the breadwinners and had full-time jobs out of the home. Because these fathers had taken care of the infants from birth on and acted as their primary attachment figures, they were included in the sample of primary caregivers. The patterns of scores of these 3 fathers, moreover, turned out to fall within the normal range in the sample. The percentages of single parents and of fathers acting as primary caregivers were representative of families in The Netherlands with children in this age group. In the sample, 38% of the primary caregivers were homemakers, and only 4% worked out of the home for more than 32 hours a week. The ages of the primary caregivers ranged from 22 to 47 years (M: 32.9 years, SD: 4.42). Their level of education ranged from low (elementary school) to high (college degree or more). The sample contained 73 firstborn infants and 56 later-born infants.”

**Inclusion MRI session – from Tyborowska et al.** ^66^

“All actively participating children from the Nijmegen Longitudinal Study on Child and Infant Development (n = 116) were approached to take part in this imaging study. Anatomical scans were obtained from participants at 14 and 17 years of age. Forty-nine at the first imaging time-point and ninety-six at the second imaging time-point agreed to participate. Participants did not have a history of psychiatric disorders or neurological illness (as indicated by parent/guardian report). Written informed consent was obtained from parents and participants during each measurement wave. The study was approved by the local ethics committee (CMO region Arnhem – Nijmegen) and was conducted in compliance with these guidelines.”

**Psychopathology at age MRI scan**

As described in Tyborowska et al. ^66^, “internalizing symptoms at age 17 were measured using the Child Behaviour Checklist (CBCL) ^70^. The CBCL is a parent-report questionnaire used to assess the frequency of emotional and behavioral problems exhibited by the adolescent in the past six months. The parent rated each behavior or symptom on a three-point Likert scale (not true, somewhat or sometimes true, very true or often true). Items from the scales anxious/

depressive, withdrawn/depressive, and somatic complaints were summed to provide a score for internalizing symptoms.”

No diagnostic interview was performed, precluding establishing diagnoses according to the DSM-5.

**Measures of temperament**

***Procedure*** *^68^* “The caregivers and children were visited in their homes for 2 hours when the child was 15 months of age. During the visit, the primary caregiver completed a Q-sort and a set of questionnaires assessing his or her ego-resiliency and attachment style, network and partner support, and child temperament. In addition, the caregiver was administered a verbal intelligence test. At the end of the visit, the caregiver and child were videotaped during the performance of four consecutive interaction tasks, lasting 3 or 4 min each. The parent was asked to have the child unlock a puzzle box, put a puppet together, do a jigsaw puzzle, and “read” a set of picture books. The parents were also told that they could help the child whenever they felt the need to.

***Questionnaire data on temperament*** The Toddler Behavior Assessment Questionnaire (TBAQ; ^71^) was used to characterize children in terms of five dimensions of temperament: activity level, pleasure, social fearfulness, anger proneness, and interest/persistence. The caregiver indicates along a 7-point scale how often he or she observed particular behaviors on the part of the child during the past month; for example, “When your child was being approached by an unfamiliar adult while shopping or out walking, how often did your child show distress or cry?” The internal consistency of the five scales was satisfactory; Cronbach’s .86 (20 items) for activity level, .82 (19 items) for pleasure, .77 (19 items) for social fearfulness, .88 (28 items) for anger proneness, and .79 (22 items) for interest/persistence.

***Behavioral observations of temperament*** from ^67^: “Within 1 week of the home visit, the parent and child visited the University laboratory. First, the child’s cognitive development was assessed. This assessment was followed by a 25-min parent–child interaction episode that was not used in the present study. After that, the 14-min ‘‘stranger/robot episode’’ was set up to measure cortisol reactivity in the infants. This episode was an adapted version of a procedure described by Mullen, Snidman, and Kagan (1993) ^72^ i.e., 3 min of free play, an encounter with a female stranger (4 min) ‘‘stranger episode’’, a confrontation with a moving robot (4 min) ‘‘robot episode’’, and again 3 min of free play. At the beginning of the session, the child was placed at the center of the room with a set of age-appropriate toys while the parent was sitting on a chair at a distance of about 1m. The parent was given a questionnaire to fill out and was instructed to respond to the child naturally, but to refrain from initiating interaction. The child played freely for 3 min. Next, an unfamiliar woman entered the room with a toy ladybird containing colorful blocks. She sat quietly for 1 min within .3 m from the child. Then she played with the ladybird and the blocks and invited the child to play with the toy (3 min). Next, she went to a cabinet in the corner of the room and placed a colorful mechanical robot (10 in. high) on the floor in front of the cabinet. The experimenter, who was sitting behind the cabinet, turned the robot’s light and frightening sounds on and off and moved the robot forwards and backwards using remote control. The unfamiliar woman invited the child to come and play with the robot (4 min). After this episode, the robot was put away and a new set of age-appropriate toys was spread out on the floor. The stranger left the room, and the child was free to play for another 3 min. The entire lab session was recorded on videotape.

To asses additional behavioral measures of infant fearfulness during the stranger/robot episode, the occurrence of three infant behaviors (adapted from Nachmias et al. ^73^) was rated from the videotapes, separately for the 4-min stranger episode and the subsequent 4-min robot episode. The behaviors were looking/referencing to parent (i.e., looking between parent and stranger with a questioning expression), proximity seeking/maintaining physical contact with parent (i.e., increasing or actively maintaining proximity to parent within one arm’s length), and crying. Each behavior was rated on a scale of 1 (not at all), 2 (sometimes), or 3 (often/most of the time) by a graduate student who was trained by the first author. Inter-observer agreement was computed on a 20% sample of randomly chosen tapes and ranged from Cohen’s k .84 to .98 for the six scores (i.e., three scores in two situations). Principal Component Analysis with Varimax rotation yielded two factors on the six behavioral ratings. The first factor, fear of stranger, had an eigenvalue of 2.98 (38.29% of the variance explained) and variable loadings of .82 for looking to parent during the stranger episode, .89 for proximity seeking during the stranger episode, and .71 for crying during the stranger episode. The second factor, fear of robot, had an eigenvalue of 1.50 (25.01% of the variance explained) and variable loadings of .83 for looking to parent during the robot episode, .75 for proximity seeking during the robot episode, and .68 for crying during the robot episode. To create two composite scores, the three scores loading on each factor were summed after standardization. These two scores, fear of stranger and fear of robot, were used as behavioral measures of infant social fearfulness. The correlation between the two composite scores was .19 ( *p* < .05).”

# Pittsburgh

**Publications:** ^74–77^

**Study design; in- and exclusion criteria**

The present set of structural MRI (sMRI) scans of third generation offspring are part of an ongoing family study that selected families through their parents’ generation. The goal of the larger longitudinal study was to contrast offspring from high and low-risk for alcohol dependence families on the basis of neurobiological and clinical status. Accordingly, offspring were followed through childhood at approximately annual intervals and through young adulthood, biennially. Extensive assessment of psychiatric disorders including alcohol and drug use information was obtained at each follow-up wave using age appropriate instruments. All participants provided consent with each visit. Children provided assent with parental consent. The study has ongoing approval from the University of Pittsburgh Institutional Review Board. Although 102 individuals were scanned at 3T before age 25, only 64 signed consent for sharing their data, and 15 of them had data on childhood temperament (peer play, see next page).

The high-risk families were identified through a proband pair of alcohol dependent brothers or pair of sisters as previously described ^74,75^. Both members of the proband pair were screened using an in-person structured interview (Diagnostic Interview Schedule; DIS ^78^) to determine the presence of alcohol dependence and other Axis I psychopathology.

Selection of control pedigrees was based on availability of a nuclear family with children between the ages of 8-18 and through parents who were screened for absence of alcohol and drug dependence using the DIS.

**Diagnostic interview**

Each child/adolescent and his/her parent were separately administered the Schedule for Affective Disorders and Schizophrenia (K-SADS)^79^ by trained, Masters’ level clinical interviewers and an advanced resident in child psychiatry at each annual evaluation. A reliable best-estimate diagnosis was obtained for all major DSM-III diagnoses at approximately yearly intervals ^74,75^. Quantity and frequency of use of commonly used substances (e.g., alcohol, cannabis, benzodiazepines, opioids) was also obtained. Young adult assessments included the Composite International Diagnostic Interview (CIDI) ^80^ and CIDI-Substance Abuse Module (CIDI-SAM)^81^, providing diagnoses for all DSM-IV diagnoses. Information concerning lifetime use of substances prior to the MRI scan was derived from the K-SADS, CIDI, and CIDI-SAM interview data. The presence of a SUD diagnosis was determined based on the outcome of the K-SADS or CIDI interviews.

Because multiple evaluations were available for each participant, the clinical evaluation closest in time but preceding the scan was chosen. Only those diagnoses that occurred prior to the scan and within 1 year of the scan were included as current diagnoses. Because neuropsychological testing that included IQ assessment occurred less frequently than the clinical interviews during childhood, only those occurring within 2 years prior to the scan were included. The educational attainment at the point in time where IQ was selected was chosen to indicate the level of education at the time of the scan.

**Measures of temperament** ^82^

**﻿*Peer play procedure (4 – 6 year olds****)*: The peer play study included 36 children who were assessed with different pairings with other children, totaling 100 sessions in all. In each pairing, a child was paired with one other child whom he/she had never met, in up to three separate sessions ^83^. Both children had mothers present within the test room who were asked to quietly observe. Observations were made during the 30-minute play session through a one-way mirrored window supplemented by cameras which provided additional views of the playroom though monitors in the observation room where coders were located. All coders met an interrater reliability criterion of *r* = 0.90 with other coders. The sessions were scored for: (1) amount of time spent proximal to the parent (within the parent's reach); (2) the amount of time staring at the other child, neither speaking nor playing with the child at the time staring occurred. Also, latency to speak, latency to touch the playroom toys and the total amount of speech were recorded ^83^. Most children participated in three sessions of peer play.

For this mega-analysis, we created a sum score of 1) average (over all sessions) total amount of time staring at the other child, 2) average amount of time spent proximal to the parent, 3) average latency to speak, as an index of cIT ^83,84^.

***Adolescent temperament measures (around time of scan)*** ^85^ As part of the longitudinal follow-up, subjects were administered the Multidimensional Personality Questionnaire (MPQ) ^86^. The MPQ provides 11 personality scales and 3 higher order scales. Assessment was completed within 1 year of the scans.

# San Raffaele

**Publications:** ^87–90^

**In- and exclusion criteria** (from ^87^)

“Subjects were drawn from a sample of 49 normally developing children who had participated in an ERP study and shyness at age 8–9 ^89^. The 49 ERP study participants had been drawn from a general population cohort (*n =* 149) assessed at age 7 for shyness ^88^. In 2007–2008, we invited all 49 children and their families to a new phase of the study, which encompassed fMRI sessions and direct psychiatric interviews: 38 (78%) accepted, 4 (8%) refused, and 7 (14%) were unavailable due to relocation. Amongst the 38 acceptant subjects, 17 withdrew for the presence of orthodontic apparels, health/family problems, or for sickness/unexpected constraints on the experiment day. This left 21 participants to this study. The procedures were accepted by the ethical committee of the participating institutions and, after complete description of the study to the subject, parental written informed consent was obtained.”

**Diagnostic interview**

“The presence of symptoms of DSM-IV childhood disorders was established by consensus of the first two authors via blinded reviews. K-SADS interviews were administered to parents while their children were undergoing fMRI on the day of the experiment. For all diagnostic categories, the K-SADS instructions ^91^ were followed and applied rigorously.” ^87^

**Measures of temperament**

As described in the baseline-paper of this longitudinal study^88^, the assessment of cIT consisted of several steps.

***Training of Teachers as Informants and Psychometric Indices***

“Before beginning the study, teachers were invited to a lecture on childhood behavioral inhibition and social anxiety disorder, and they also participated in a hands-on seminar on the format and wording of the questionnaire. All items were presented in detail and examples of behaviors that applied to items were provided. Further training of teachers was provided through question times and educational papers on childhood behavioral inhibition and social

anxiety disorder. The questionnaire sought to identify (1) symptoms of possible social anxiety disorder proper and (2) temperamental disposition to behavioral inhibition as possible correlates and external validators of social anxiety symptoms ^92,93^. Three different scales were used in the questionnaire: (1) the Liebowitz Social Anxiety Scale adapted for children (LSAS) ^94,95^ to evaluate symptoms of fear and avoidance of social situations using DSM-IV criteria of social anxiety disorder, (2) the Shyness-to-the-Unfamiliar (SU) Scale ^96,97^ to evaluate temperamental shyness and the modality of approach to the unfamiliar, and (3) the Harm Avoidance (HA) scale of Cloninger’s Junior Temperament and Character Inventory, Parent version ^98^, to measure temperamental disposition toward avoidant behaviors in the face of uncertainty.

***LSAS Scale***

The LSAS is usually administered to children or to informants by an interviewer ^94^, but for the purposes of this study the wording was adapted to allow the scale’s completion by trained teachers who acted as informants. The original LSAS includes 24 items rated 0 (“no fear/never avoids”) to 3 (“severe fear/usually avoids”), but for our purposes items 5, 9, and 21

(“talking/answering telephone” and “urinating in public restroom”) were excluded because teachers could not rate them adequately, so that in our study the LSAS ranged from 0 to 63. Previous studies showed validity and clinical usefulness of the LSAS in (1) assessing

the reduction of fear and avoidance of social contests in school-based behavioral treatments for social anxiety disorder in adolescents ^95^ and (2) clarifying the presence of social

anxiety disorder as the salient clinical feature of childhood selective mutism ^94^

***SU Scale***

The SU assesses the initial approach to/withdrawal from unfamiliar people: it encompasses six items (e.g., “readily plays with a new child,” “avoids new guests/visitors”) rated 0 to 5 (from “almost never” to “almost always”), and ranges from 0 to 30. Behavioral inhibition evaluated by the SU scale has revealed good temporal stability (r ∼ 0.5) from age 4.5 to age 7 ^99^. The SU scale has been linked with some physiological correlates of behavioral inhibition and generally has predicted familial social phobia; subjects rated as behaviorally inhibited with the SU scale tend to have a higher heart rate ^99^. Furthermore, in a community study of schoolchildren, an association was found between children’s higher rates on the SU and mothers’ heightened risk for social phobia ^96^.

***HA Scale***

The HA scale encompasses 22 true/false items and thus ranges from 0 to 22; it is organized into four subscales: Fear of Uncertainty, Worry and Pessimism, Shyness With Strangers, and Fatigability. As with the LSAS, the HA parent version was modified to allow teachers’ use. Harm avoidance has a heritability of about 0.5 ^98^, is relatively stable from childhood to adult life ^100^, and heightens the risk of developing symptoms of anxiety/depression for people with extreme scores on the HA scale ^101^.

The teachers were asked to base their judgment on the instructions received from our group at seminars and on direct observation. On the basis of an anonymous review of pupils’ individual reports available from the school archive, children were excluded from assessment if they (1) had joined the class less than 6 months earlier, (2) displayed mental/physical handicaps that would require special attention, such as a remedial teacher, and (3) revealed learning disabilities and/or overt attention difficulties. This left 149 subjects (mean age 7.5 ± 0.5 years) who underwent an expression discrimination trial that was administered at school by four psychologists trained in the evaluation of childhood behavior.”

For the present mega-analysis, we will use an empirical composite index of cIT encompassing latency of first spontaneous comment, items from the Stevenson- Hinde and Glover Shyness to the Unfamiliar, Cloninger's Harm Avoidance and the Liebowitz Social Anxiety Scale adapted for children ^89^.

# SDAN

**Publications:** ^102–104^

**In- and exclusion criteria (from** ^102,104^**); diagnostic information**

The sample comprised healthy volunteers and youth diagnosed with an anxiety disorder, disruptive mood dysregulation disorder, or ADHD by licensed clinicians using the Kiddie Schedule for Affective Disorders and Schizophrenia (K-SADS) ^91^. Exclusion criteria were neurological disorders, autism and bipolar spectrum disorders, psychosis, substance use, MRI contraindications, and Full Scale IQ below 70. Anxiety was assessed by using the parent- and youth-reported ratings of the five subscales of the Screen for Child Anxiety Related

Disorders (SCARED) ^12^.

**Measures of temperament**

Behavioral Inhibition Questionnaire ^105^.

# Stony Brook Temperament Study

**Publications:** ^31,106–110^

**In- and exclusion criteria** (described in ^110^)

For this longitudinal study, “participants were recruited from the community utilizing

commercial mailing lists, screened for any major medical conditions, and required to have at least one English-speaking biological parent. Exclusionary criteria included any developmental disabilities, metal or electronic implants, a history of head trauma, or use of medications known to affect brain functioning (e.g., antihistamines, pain killers). Participants were oversampled based on their temperamental negative emotionality, low positive emotionality, or behavioral inhibition, assessed observationally when they were 3 years old (see ^111^ ). This oversampling was done as the broader goal of the study was to understand early childhood risk factors for later depressive and anxiety disorders, for which high negative emotionality, low positive emotionality, and high behavioral inhibition are risk factors (see Olino et al. ^112^ for details). Negative and positive emotionality as well as behavioral inhibition were assessed via the Laboratory Temperament Assessment Battery (LabTAB) ^46^, which involves a standardized set of tasks designed to elicit children’s bodily, vocal, and facial expressions of a range of emotions (see ^113^).”

The MRI sample was a subsample of the age 3 sample and were selected on age 3 temperament traits based on the LabTAB at age 3.

**Diagnostic interview**

Parents completed the Preschool Age Psychiatric Assessment interview at ages 3 and 6 ^114^; parents and youth completed the K-SADS at ages 9, 12, and 15.

**Measures of temperament**

(Quoted from ^113^): “Each child and a parent (95.0 % mothers) visited the laboratory for a 2-hour observational assessment of temperament that included a standardized set of 12 episodes selected to elicit range of temperament-relevant behaviors. Eleven episodes were from the Laboratory Temperament Assessment Battery (Lab-TAB) ^46^ and one was adapted from a Lab-TAB episode. Using an independent sample, we previously reported moderate stability of laboratory ratings of temperament from ages 3 to 7 (*r* = .46 and .45 for positive emotionality and negative emotionality, respectively), and moderate concurrent and longitudinal

associations between Lab-TAB ratings and home observations ^115^. Each task was videotaped through a one-way mirror and later coded. To prevent carryover effects, no episodes presumed to evoke similar affective responses occurred consecutively and each episode was followed by a brief play break to allow the child to return to a baseline affective state. The parent remained in the room with the child for all episodes except Stranger and Box Empty, but was instructed not to interact with the child (except in Pop-Up Snakes), and was seated facing at a right angle from the experimenter and child and given questionnaires to complete.

The episodes, in order of presentation, were: (1) Risk Room. Child explored a set of novel

and ambiguous stimuli, including a Halloween mask, balance beam, and black box; (2) Tower of Patience. Child and experimenter alternated turns in building a tower. The experimenter took increasing amounts of time before placing her block on the tower during each turn; (3) Arc of Toys. Child played independently with toys for five minutes before the experimenter asked the child to clean up the toys; (4) Stranger Approach. Child was left alone briefly in the room before a male accomplice entered, speaking to the child while slowly walking closer; (5) Make that Car Go. Child and experimenter raced remote-controlled cars; (6) Transparent Box. Experimenter locked an attractive toy in a transparent box, leaving the child alone with a set of non-working keys. After a few minutes, the experimenter returned and told the child that she had left the wrong set of keys. The child used the new keys to open the box and play with the toy; (7) Exploring New Objects. Child was given the opportunity to explore a set of novel and ambiguous stimuli, including a mechanical spider, a mechanical bird, and sticky soft gel balls; (8) Pop-up Snakes. Child and experimenter surprised the parent with a can of potato chips that actually contained coiled snakes; (9) Impossibly Perfect Green Circles. Experimenter repeatedly asked the child to draw a circle on a large piece of paper, mildly criticizing each attempt; (10) Poppin Bubbles. Child and experimenter played with a bubble-shooting toy; (11) Snack Delay. Child was instructed to wait for the experimenter to ring a bell before eating a snack. The experimenter systematically increased the delay before ringing the bell; and (12) Box Empty. Child was given an elaborately wrapped box to open under the impression that a toy was inside. After the child discovered the box was empty, the experimenter returned with several toys for the child to keep.

***Coding Procedures*** Behavioral inhibition (BI) was coded using an approach that was similar to most previous studies ^116^. The three episodes specifically designed to assess BI (Risk Room, Stranger Approach, Exploring New Objects) were divided into 20 or 30 second epochs, and a series of affective and behavioral codes were rated for each epoch ^46^. Within each epoch, a maximum intensity rating of facial, bodily, and vocal fear was coded on a scale of 0 (absent) to 3 (highly present and salient). Based on previous studies using the Lab-TAB ^117^, BI was computed as the average standardized ratings of latency to fear (reversed); and facial, vocal, and bodily fear (Risk Room, Stranger Approach, and Exploring New Objects); latency to touch objects; total number of objects touched (reversed); tentative play; referencing the parent; proximity to parent; referencing the experimenter; and time spent playing (reversed) (Risk Room and Exploring New Objects); startle (Exploring New Objects); sad facial affect (Exploring New Objects and Stranger Approach); and latency to vocalize; approach towards the stranger (reversed); avoidance of the stranger; gaze aversion; and verbal/nonverbal interaction with the stranger (reversed; Stranger Approach).”

At age 3, a parent also completed the Behavioral Inhibition Questionnaire ^26^.

For the present mega-analysis, we will use a sum score (log-transformed) from 3 Kagan-like tasks in Goldsmith's Lab-TAB as an index of cIT.

# TOTS

**Publications:** ^118,119^

**In- and exclusion criteria** ^118,120^

This study concerns a longitudinal project. The selection of participants was as follows (described by Hane et al., 2008): “Families identified via commercially available mailing lists were sent a letter about the project and were asked to complete a form and send it back to the laboratory. Interested mothers of developmentally healthy infants were scheduled for a laboratory visit between their infant’s 15th and 17th weeks.

***Four-Month Selection.*** 779 infants were screened for degree of reactivity to visual and

auditory stimuli at four months (see ^42,121^). Infant behavior during the reactivity paradigm was subsequently coded as follows: A motor reactivity score was obtained by summing the frequencies of arm waves, arm wave bursts (several waves in rapid succession), leg kicks, leg kick bursts, back arches and hyper extensions throughout the paradigm. A negative affect score was derived by summing the frequencies of fussing and crying and a positive affect score was obtained by summing the frequencies of smiling and positive vocalizations.

The first 100 infants screened were used as a criterion group, i.e., their negative, positive, and motor reactivity scores were used to set the selection criteria for all subsequent infants as

follows: Infants who scored above the criterion group mean on both negative affect and motor

arousal and below the mean on positive affect served as the negatively reactive (NR) group (*n* = 75). Infants who scored above the criterion group mean on both positive affect and motor arousal and below the mean on negative affect served as the positively reactive (PR) group (*n* = 73). Eighty-six infants who did not meet the criteria for either temperament group served as the control sample. Four reliable raters coded the four-month reactivity paradigm, with pairs of coders achieving intraclass correlation coefficients ranging from .80 to .92. A MANOVA comparing the three temperament groups on the three reactivity dimensions was significant (*p*< .001). The NR group manifested significantly more negative affect than both the PR and control groups (*F* (2, 231) = 75.08, *p* < .001; Tukey’s HSD both *p*’s < .001). The PR group displayed significantly more positive affect than the NR and the control groups (*F* (2, 231) = 41.94, *p* < .001; Tukey’s HSD both *p*’s < .001). The control group showed significantly less motor activity than both the NR and PR groups (*F* (2, 231) = 51.17, *p* < .001; Tukey’s HSD both *p*’s < .001). Based on four-month temperament group status, 278 infants were invited to continue participation. 268 children returned to the laboratory at 2 and 3 years of age for BI assessment.^42^

At ages 10 and 12, eligible children were invited to participate in brain imaging visits. Brain imaging visits were conducted at the National Institute of Mental Health as part of the longitudinal assessment. Participants were excluded if they were taking any psychotropic medications at the time of scanning. However, subjects on psychostimulant medications who could tolerate a 24 -h medication-free period prior to scanning were included. Children were also deemed ineligible to participate in imaging visits if they had an MRI contraindication (i.e., metal in their body). Parental consent was obtained prior to all visits and child assent was obtained prior to 10 and 12 year visit.”

**Diagnostic interview**

Psychopathology around the time of scan was assessed using the Kiddie Schedule for Affective Disorders and Schizophrenia (K-SADS). Trait anxiety symptoms were measured using the SCARED, a reliable child- and parent-report questionnaire with 42 items ^30,122^.

**Measures of temperament**

At age nine months, data were collected using 6 LabTAB tasks: 2 anger/frustration tasks (arm restraint, toy barrier), 2 fear tasks (masks, unpredictable toy), and 2 joy tasks (peek-a-boo, puppets) ^123^. Furthermore, inhibited behavior to novel stimuli was coded at 24 and 36 months ^42^. Behavioral scores were standardized at each time point.

For the present study, we will use the ‘BI-classic-24’ index, composed of scores on the stranger, robot and tunnel episodes of the Laboratory Temperament Assessment Battery – preschool version ^46^.

# Vanderbilt - children

**Publications:** ^124^ and unpublished

**In- and exclusion criteria**

Quoted from Clauss et al ^124^: “Consistent with the extreme discordant phenotypes approach ^125^, we compared inhibited children and uninhibited children at the extreme ends to maximize our chances of identifying differences. To obtain pure risk groups (not confounded by existing disorders), children were excluded from the study for having any current or past psychiatric

diagnoses, as measured by the Kiddie-Schedule for Affective Disorders and Schizophrenia–Present and Lifetime Version (KSADS-PL) ^91^ or having received treatment for anxiety symptoms. Children were also excluded if they had cognitive deficits that might affect

task performance (developmental delay, repeating a grade, or receiving special assistance in school), contraindications to MRI scanning, or factors that might affect blood oxygen level-dependent (BOLD) signal (psychotropic medications, history of head injury, major medical or neurological conditions). Intelligence quotient (IQ) was assessed using the Kaufman Brief Intelligence Test ^126^. Handedness was assessed using the Edinburgh Handedness Inventory ^127^.

Participants were recruited from the Vanderbilt University Medical Center and surrounding community using flyers, e-mails, and research recruitment databases. Advertisements were for children who were “quiet,” “cautious,” “shy,” “outgoing,” and general recruitment for a study on “temperament and brain function.” Before the first study visit, parents completed a brief online screening, including the Behavioral Inhibition Questionnaire-Parent (BIQ-P) ^26^, a validated measure of childhood inhibited temperament, which shows convergent validity with behavioral measures and other measures of social inhibition ^27,31^.

Although 4 questions in the questionnaire refer to younger age groups, these questions were highly correlated with other items in the scale and therefore were retained as written. Children were selected based on a temperament score plus or minus 1 standard deviation from the mean based on published norms (inhibited >123; uninhibited <59)^26^; these norms were similar to those identified in children and adolescents 4 to 15 years of age39 and those used in a

recent similar neuroimaging study ^16^.”

**Diagnostic interview and psychiatric symptom measures**

Kiddie-Schedule for Affective Disorders and Schizophrenia–Present and Lifetime Version (KSADS-PL) ^91^ - see above. To further characterize participants, both parents and children reported on a number of psychiatric symptom measures, including, among others the Screen for Child Anxiety-Related Disorders ^122^ and the Children’s Depression Inventory ^128^.

**Measures of temperament**

Children completed a self-report of temperament, the Behavioral Inhibition Questionnaire-Child (BIQ-C). ^27^. The total score will be used as an index of cIT in the present mega-analysis.

# Vanderbilt – young adults

**Publications:** ^129–133^

**In- and exclusion criteria**

The sample in this mega-analysis consist of participants from multiple previously published studies. In general, ﻿as described in Clauss et al. ^132^, subjects were recruited by seeking individuals ages 18–25 who were “extremely shy or outgoing.” Consistent with prior studies ^130,131,134^, individuals with an extreme inhibited or extreme uninhibited temperament were identified using the Adult Self-Report of Inhibition (ASRI) and the Retrospective Self-Report of Inhibition (RSRI) ^65^. Subjects were selected for having a stable temperament (i.e., being extremely inhibited or extremely uninhibited as both an adult and a child), defined by scores on both the ASRI and RSRI that were greater than one standard deviation from published means. Other inclusion criteria included: passing an MRI safety screen, being free of psychoactive medications within the past 6 months, having no history of brain trauma, and having no psychiatric illness (based on clinical interview), except anxiety disorders in the inhibited temperament group. Inhibited subjects who met criteria for a current or past anxiety disorder were not excluded.

**Diagnostic interview**

Psychiatric diagnosis was assessed by a trained clinical interviewer using the Structured Clinical Interview for DSM-IV ^135^.

**Measures of temperament**

**﻿**To focus on a stable trait and ensure that valid groups were identified, inhibited temperament was assessed retrospectively (childhood) and currently using the Retrospective Self-Report of Inhibition (RSRI) and the Adult Self-Report of Inhibition (ASRI) ^65^, respectively. ﻿Both questionnaires have excellent reliability (Cronbach’s alpha = 0.79 for the RSRI and 0.78 for the ASRI), demonstrate convergent validity ^65,136^ and minimize self-report bias by focusing on reports of concrete behaviors in specific situations instead of subjective feelings^137^. In the sample for the current mega-analysis (*n*  = 150), scores on the RSR and ASRI were highly correlated (*r*  = 0.91, p < 0.001); we will use the total scores on the RSRI as index of cIT.

# Western University

**Publications:** ^138–144^

**In- and exclusion criteria**

As described in Vandermeer et al., 2020 ^142^, children (*n* = 87) and their mothers were recruited from a larger longitudinal study of children’s depression risk (*n* = 409) that began when children were 3-year-olds. At baseline, children with major medical or psychological problems were excluded, and typical cognitive development was verified using the Peabody Picture Vocabulary Test-Fourth^145^. For the current study, children were recruited from the larger longitudinal sample based on maternal history of depression (MH+) drawn from data collected at a previous round of data collection for this study ^138^. Children were considered high-risk based on a maternal history of recurrent major depression (*n* = 26), or a maternal lifetime history of a single major depressive episode and a serious anxiety disorder (i.e., any anxiety disorder except a specific phobia; *n* = 3). Low-risk children had no maternal history of major depression or anxiety disorder. From this sample, 237 families were contacted (58 MH+). Children with any contraindications to the MRI scan (e.g., braces, metallic objects implanted in the body, claustrophobic) were deemed ineligible, leaving a pool of 231 families, from which 110 families agreed to participate (36 MH+). Children from these families were screened as described in the following section to ensure the absence of current or lifetime depressive disorder^[2](https://www.sciencedirect.com/science/article/pii/S2213158220302321#fn2)^. Eighty-seven children (29 MH+; 49 boys) participated in the MRI session.

**Diagnostic interview**

Children were administered the K-SADS-PL and completed self-reported symptom and severity measures, including the Children’s Depression Inventory 2nd Edition (CDI; ^146^; *α* = 0.83) and the Youth Self-Report ^147^ with the help of trained graduate students in clinical psychology.

**Measures of temperament**

A detailed description of the assessment of temperament is provided in previous publications on the study, for example Vandermeer et al.^140^ and Liu et al.^138^ . Of interest for the present work is the baseline measurement of the study, in which temperament was assessed during a standardized lab visit based on the Laboratory Temperament Assessment Battery (Lab-TAB; ^46^) and an age-adapted version of the Lab-TAB ^115^. During this lab visit, children participated in tasks drawn directly from the preschool-aged version of the Lab-TAB. The assessment of behavioral inhibition (BI) at age three consisted of three Lab-TAB tasks: Risk Room, Stranger Approach, and Exploring New Objects, as described in ^138,140^. Age 3 BI scores were a composite score based on the average of z-scores for coded variables for the three tasks. These procedures for computing BI composite scores are consistent with other studies using observational coding (e.g., ^107,148^). These age 3 scores will be used as an index of cIT in the present mega-analysis.

# Wisconsin Twin Project

**Publications:** ^149–151^

**Study design** (from ^150^)

“The Wisconsin Twin Project sample is based on birth-record based cohorts of twins born in the state of Wisconsin during the years 1989–2004 ^151–154^. After nearly 30 years, the research program encompasses a series of longitudinal studies that span infancy to early adulthood. Twin family recruitment and early results were covered in prior overviews of the project ^151,152^. Briefly, initial contact was attempted with a mailed letter and contact form. Contact was maintained with multiple phone numbers, email addresses, a toll-free phone number and secondary contact information from a family friend or relative. Sample retention efforts included newsletters and a website devoted to participant communication. Web-based tracing

methods (e.g., public court records) were used to locate families with whom we had lost contact. The University of Wisconsin Survey Center also provided tracing services. All of these procedures helped maintain the research sample longitudinally. The research is conducted at the University of Wisconsin– Madison’s Waisman Center and the Department of Psychology

(https://goldsmithtwins.waisman.wisc.edu/). Procedures in studies under the Wisconsin Twin Project were approved by University of Wisconsin–Madison Internal Review Boards and comply with the Helsinki Accords of 1975, as revised in 2008.”

In the present mega-analysis, we will use data from the RDoC twin study (participants < 13 years of age), as these data are available through the National Institute of Mental Health Data Archive (NDA). As summarized in Schmidt et al. ^150^, “the RDoC twin study used longitudinal an quantitative genetic approaches to establish developmental antecedents and neural substrates for the RDoC positive valence systems (e.g., anticipatory positive affect and contentment) an negative valence systems (e.g., acute fear, potential threat/anxiety, frustrative non-reward and loss). The RDoC twin study aimed to (a) establish distinctiveness, stability and external validity of each RDoC construct during childhood and adolescence; (b) investigate the relationship between brain structure and function (via MRI) and concurrent and longitudinal RDoC measures; and (c) utilize the MZ difference design to highlight early environmental contributions to later brain structure and function. In the MRI analyses, we focused more on white matter microstructure and on resting state and task-related functional measures (i.e., circuitry and networks) than on gray matter structure. The RDoC twin study enrolled 518 MZ (56%) and dizygotic (DZ) twin individuals (mean age = 17.4 years, SD = 2.2 years). Approximately 70% were under 18 years of age at the time of assessment. Parents (88% mothers) of adolescents completed surveys. Data collection concluded in early 2019.”

**Diagnostic interview**

Not available.

**Measure of temperament**

In the present analysis, we will use the temperament assessments which took place during middle childhood (age 7), as described in ^155^. “The four-hour, in-home assessment involved additional parent questionnaires and interviews, child interviews, observer ratings, and the Lab-TAB. Data collection for this middle childhood phase took place across more than five years.

**Laboratory Temperament Assessment Battery***.* Lab-TAB ^156^ is a laboratory-based behavioral assessment that comprises multiple episodes designed to tap observable elements of temperament dimensions. Lab-TAB was administered during the childhood home visit and was modified slightly for use in homes ^157^. During Lab-TAB administration, children’s behavior was videotaped and later coded by individuals blind to other information about the child. Individual raters did not rate both twins from the same family. 10% of the videos were rated by a master coder, and agreement between master coder and the other coder (Kappa > 0.70) was required. Each Lab-TAB episode (3-10 minutes duration), provided multiple responses scored in 5-30 second epochs or in discrete trials. Parameters included latency to first response, occurrence of a target response within an epoch or trial (mean response), and the magnitude or intensity of a target response (peak response). In general, positivity was coded as absence/presence (0/1) while facial, bodily, and vocal angry, sad, and fearful responses were coded on a 0-2 or 0-3 scale. For detailed descriptions of each episode and of scoring procedures see the Lab-TAB manual ^158^.

In **Storytelling**, the child stands in front of multiple child testers and is asked to talk about what they did the prior day, with least one prompt given by the child tester ^155^. In **Stranger approach**, social interaction with an unfamiliar adult wearing hat and sunglasses is investigated ^157^.

**Post-visit observer ratings** (from Moore et al. ^155^)*. “*Two child testers from each middle childhood home visit independently completed post-visit ratings for each twin on 28 items related to child behavior, where “1” indicates the absence of the characteristic or behavior and “5” describes an extreme reaction. Behavior was observed throughout the visit, including times before, between, and after administration of Lab-TAB episodes. Some items include modified content from the Behavior Rating Scales (BRS) from the Bayley Scales of Infant Development. Child tester ratings were averaged for each item; item-specific correlations between raters ranged from .38 to .49.”

**Additional questionnaire data (parental report) on inhibited temperament**

**MacArthur Health and Behavior Questionnaire***:* inhibition scale from the MacArthur Health and Behavior Questionnaire (HBQ; ^159^). From Moore et al^155^: “Parents rated their child’s behavior over the past six months using a 3-point scale (0 = rarely, 2 = certainly applies). Internal consistency reliability (alpha) for age 7 HBQ subscales ranged from .67 to .84 for mother-report and from .62 to .85 for father-report. Mother and father scores were moderately and significantly correlated (age 7 *r*s ranged from .27 to .53, all *p*s < .001) and were mean-averaged into a single parent-report score at each age.”

**Children’s Behavior Questionnaire (CBQ)**

An abridged 80-item version of the CBQ was completed by both parents. As described in Gagne et al.^157^, the CBQ requires parents to judge their children’s reactions to a variety of situations over the last 6 months (e.g., “Can lower his/her voice when asked to do so”) and is appropriate for children from 3 years to 7 years of age ^160^. Each item is rated on a 1–7 scale, with 1 indicating the reaction is extremely untrue of the child and 7 indicating that the reaction is extremely true. CBQ scores have shown high internal consistency, parental agreement, and convergent validity with socialization-relevant traits ^160^ and have been used in numerous studies with a wide range of empirical correlates. The eight CBQ scales that we used were selected for overlap with temperament dimensions assessed in the Lab-TAB, and each CBQ scale had 10 items. Estimates of internal consistency for each CBQ scale were as follows: Anger (α = .78), Fear (α = .73), Shyness (α = .92), Sadness (α = .63), Approach (α = .74), Activity Level (α = 73), Attentional Focusing (α = .78), and Inhibitory Control (α = .82).”

For the present mega-analysis, we used a sum-score of the behavioral observations related to inhibited behavior (ratings on Approach and Shyness from the home visit by 2 observers and the scores from videotaped reactions to the “Conversation with a Stranger” episode of Lab-TAB). This sum-score correlated significantly with the parental reports on inhibited temperament (correlation sum-score with HBQ-Mother (inhibition): *r* = 0.28, *p* < 0.001; with HBQ-Father (inhibition): *r*  = 0.18, *p* = 0.004; with CBQ-Mother (shyness): *r* = 0.29, *p* < 0.001; with CBQ-Father (shyness): *r*  = 0.30, *p*  < 0.001).

**Supplemental Tables**

**Supplemental Table 1 STROBE checklist case-control studies**

**Supplemental Table 2 Scanner characteristics per sample**

**Supplemental Table 3 Clinical characteristics per sample**

**Supplemental Table 4 Overview of included independent variables per sample**

**Supplemental Table 1 STROBE Statement—Checklist of items that should be included in reports of *case-control studies***

|  | Item No | Recommendation | Location in manuscript |
| --- | --- | --- | --- |
| **Title and abstract** | 1 | (*a*) Indicate the study’s design with a commonly used term in the title or the abstract | Title |
|  |  | (*b*) Provide in the abstract an informative and balanced summary of what was done and what was found | Synopsis |
| Introduction | | |  |
| Background/rationale | 2 | Explain the scientific background and rationale for the investigation being reported | Synopsis and Registered Report-Introduction |
| Objectives | 3 | State specific objectives, including any prespecified hypotheses | Registered Report-Introduction |
| Methods | | |  |
| Study design | 4 | Present key elements of study design early in the paper | Registered Report-Methods |
| Setting | 5 | Describe the setting, locations, and relevant dates, including periods of recruitment, exposure, follow-up, and data collection | Registered Report-Methods |
| Participants | 6 | (*a*) Give the eligibility criteria, and the sources and methods of case ascertainment and control selection. Give the rationale for the choice of cases and controls | Registered Report-Methods |
|  |  | (*b*) For matched studies, give matching criteria and the number of controls per case | *Not applicable* |
| Variables | 7 | Clearly define all outcomes, exposures, predictors, potential confounders, and effect modifiers. Give diagnostic criteria, if applicable | Registered Report-Methods |
| Data sources/ measurement | 8* | For each variable of interest, give sources of data and details of methods of assessment (measurement). Describe comparability of assessment methods if there is more than one group | Registered Report- Supplemental Methods |
| Bias | 9 | Describe any efforts to address potential sources of bias | Registered Report-Methods |
| Study size | 10 | Explain how the study size was arrived at | Registered Report-Methods |
| Quantitative variables | 11 | Explain how quantitative variables were handled in the analyses. If applicable, describe which groupings were chosen and why | Registered Report-Methods |
| Statistical methods | 12 | (*a*) Describe all statistical methods, including those used to control for confounding | Registered Report-Methods |
|  |  | (*b*) Describe any methods used to examine subgroups and interactions | Registered Report-Methods |
|  |  | (*c*) Explain how missing data were addressed | Registered Report-Methods |
|  |  | (*d*) If applicable, explain how matching of cases and controls was addressed | *Not applicable* |
|  |  | (*e*) Describe any sensitivity analyses | Registered Report - Methods |

| Results | | |  |
| --- | --- | --- | --- |
| Participants | 13* | (a) Report numbers of individuals at each stage of study—eg numbers potentially eligible, examined for eligibility, confirmed eligible, included in the study, completing follow-up, and analysed |  |
|  |  | (b) Give reasons for non-participation at each stage |  |
|  |  | (c) Consider use of a flow diagram |  |
| Descriptive data | 14* | (a) Give characteristics of study participants (eg demographic, clinical, social) and information on exposures and potential confounders |  |
|  |  | (b) Indicate number of participants with missing data for each variable of interest |  |
| Outcome data | 15* | Report numbers in each exposure category, or summary measures of exposure |  |
| Main results | 16 | (*a*) Give unadjusted estimates and, if applicable, confounder-adjusted estimates and their precision (eg, 95% confidence interval). Make clear which confounders were adjusted for and why they were included |  |
|  |  | (*b*) Report category boundaries when continuous variables were categorized |  |
|  |  | (*c*) If relevant, consider translating estimates of relative risk into absolute risk for a meaningful time period |  |
| Other analyses | 17 | Report other analyses done—eg analyses of subgroups and interactions, and sensitivity analyses |  |
| Discussion | | |  |
| Key results | 18 | Summarise key results with reference to study objectives |  |
| Limitations | 19 | Discuss limitations of the study, taking into account sources of potential bias or imprecision. Discuss both direction and magnitude of any potential bias |  |
| Interpretation | 20 | Give a cautious overall interpretation of results considering objectives, limitations, multiplicity of analyses, results from similar studies, and other relevant evidence |  |
| Generalisability | 21 | Discuss the generalisability (external validity) of the study results |  |
| Other information | | |  |
| Funding | 22 | Give the source of funding and the role of the funders for the present study and, if applicable, for the original study on which the present article is based |  |

*Give information separately for cases and controls.

**Supplemental Table 2 Scanner characteristics per sample**

| **Sample** | **Scanner type** | **Field-strength** | **Structural MRI scan** |
| --- | --- | --- | --- |
| Brains study | Siemens Trio  Siemens Prismafit | 3 T | High-resolution T1-weighted structural scans with a magnetization prepared gradient echo sequence (MP-RAGE) (176 1 mm slices, TR = 1700, TE = 2.01, FA = 9°, FOV = 256 mm, voxel size = 1 × 1 × 1 mm; 256 × 256matrix, T1 = 850 ms). |
| Brazilian High Risk Cohort | Signa HDx (São Paulo) GE Signa HD (Porto Alegre) | 1.5 T | T1-weighted structural MR images were acquired with the following parameters: TR=10.916 ms, TE=4.2 ms, slice thickness=1.2 mm, FA =15°, matrix size=2563192, FOV=245 mm, max=156 slices). |
| Cohort 3 / 4 | GE Healthcare MR750 | 3 T | High-resolution, T1-weighted structural imaging sequence (MPRAGE; sagittal acquisition; 176 slices; 1 mm^3^ isotropic voxels; 256 * 256 matrix; flip angle = 7°; TR = 7.7 ms; TE = 3.42 ms; TI = 425 ms). |
| Generation R - sample with behavioral observations and | GE MR750 | 3 T | ﻿T1-weighted inversion recovery fast spoiled gradient recalled (IR-FSPGR) sequence with the following parameters: TR = 8.77 ms, TE = 3.4 ms, TI = 600 ms, FA = 10°, matrix 220*220, slice thickness 1.0 mm, in-plane resolution (mm) 1.0 mm^2^ |
| Generation R - sample with questionnaire data |  |  |  |
| Maryland-PAX | Siemens Magnetom TIM Trio | 3 T | Sagittal T1-weighted anatomic images with MPRAGE sequence (TR = 2400 ms; TE = 2.01 ms; TI = 1060 ms; FA = 8°; sagittal slice thickness = 0.8 mm; in-plane = 0.8 x 0.8 mm; matrix = 300x320; FOV= 240x256). |
| Maryland-TAX | Siemens Magnetom TIM Trio | 3 T | High-resolution anatomical (T1-weighted) images with a magnetization-prepared, rapid-acquisition, gradient-echo sequence (TR = 1900 ms; TE = 2.32 ms; TI = 900 ms; FA = 9°; sagittal slice thickness = 0.9 mm; voxel size in plane = 0.449 × 0.449 mm; matrix = 512 × 512; FOV = 230 × 230). |
| Nijmegen Longitudinal Study | Siemens Magnetom Trio or PRISMA | 3 T | MPRAGE sequence (TR = 2300 ms; TE = 3.03 ms; 192 sagittal slices; 1.0 x 1.0 x 1.0 mm voxels; FOV = 256 mm). |
| Pittsburgh | Siemens Trio scanner | 3 T | 7-min 3D T1-weighted Magnetization Prepared Rapid Gradient Echo Imaging (MPRAGE) sequence (TR = 2300 ms, TE = 2.98 ms, FA = 9°, field of view FOV=240 mm, acquisition matrix=240 x 256, in-plane resolution 1.0x1.0 mm^2^, yielding 160 transversal slices with a thickness of 1.2 mm). |
| San Raffaele | Philips Achieva | 3 T | T1-weighted scan, 150 axial slices, resolution 1mm x 1mm x 1mm |
| SDAN | General Electric MR750 | 3 T | Whole-brain, high-resolution, T1-weighted anatomical scan (MPRAGE; 176 axial slices, 256 x 256 matrix,1 mm ^3^ isotropic slices; FA = 7°, FOV = 220 mm; TR = 7.7ms, TE = 3.42s) |
| Stony Brook Temperament Study | Siemens Trio | 3 T | T1-weighted high resolution structural images with the magnetization prepared rapid gradient echo (MPRAGE) sequence: slices = 176, slice thickness =1 mm, TR = 2400 ms, TE = 3.16 ms, FA=8°, matrix size = 256 × 256, FOV=256 × 256 mm, resolution=1 × 1 × 1 millimeters |
| TOTS | General Electric MR750 | 3 T | High-resolution T1-weighted whole-brain volumetric scan with a high-resolution magnetization prepared gradient echo sequence (MPRAGE; TE = min full; TI = 425 ms; FA = 7°; FOV = 256 mm; matrix = 256 x 256; in plane resolution = 1 x 1x 1mm). |
| Vanderbilt - children | Philips | 3 T | T1-weighted structural data were acquired using the following parameters: 256 mm field of view (FOV), 170 slices, 1-mm slice thickness, 0-mm gap, 2-second TR, 22- millisecond TE, 90 ° flip angle, 1.8 SENSE factor, 240-mm FOV, 3 *3 mm in-plane resolution. |
| Vanderbilt - young adults | Philips | 3 T | ﻿High resolution T1-weighted anatomical images (256mm FOV, 170 slices, 1-mm slice thickness, 0-mm gap) |
| Western University | Siemens Trio | 3 T | T1-weighted 3D magnetization prepared rapid gradient echo (MPRAGE) sequence (1* 1 * 1 mm), voxel size, TR = 2300 ms, TE = 2.98 ms, FOV = 256 mm), 192 slices. |
| Wisconsin Twin Project - RDoC twin study | GE SIGNA (Discovery MR750) scanner | 3 T | T1-weighted structural images (1 mm3 voxels) were also acquired axially with an isotropic 3D Bravo sequence (TE = 3.2 ms, TR = 8.2 ms, TI = 450 ms, flip angle = 12◦) |

**Abbreviations:** FA: flip angle; FOV: field of view; T: Tesla; TE: echo time; TI: inversion time; TR: repetition time.

**Supplemental Table 3a Clinical characteristics per sample – psychopathology at (around) time of scan**

|  |  |  | **Psychopathology based on clinical interview (current / lifetime but not current)** | | | | | | | | | | | |  | |
| --- | --- | --- | --- | --- | --- | --- | --- | --- | --- | --- | --- | --- | --- | --- | --- | --- |
|  |  |  | **Anxiety disorders** | | | | | | **Other psychopathology** | | | | | | **Psychotropic medication at time scan** | |
| **Sample** | **Clinical interview** | ***n* (*n* female)** | **SAD** | **PD** | **AG** | **GAD** | **SPH** | **Other anxiety disorders** | **MDD** | **OCD** | **PTSD** | **SUD** | **Other** | | ***n*** | **Specification medication** |
| Brains study | C-DISC 4 administered to parents | 130 (72) | 4 SAD,  3 social phobia / *na* | *na* | *na* | 4 / *na* | *na* | 0 | 0 / *na* | *na* | *na* | *na* | 13 current ADHD | | 9 | *n* = 2 SSRI/SNRI *n* = 7 medication for ADHD |
| Brazilian High Risk Cohort | ﻿Development and Well-being Assessment (DAWBA) | 678 (290) | 7 / *na* | 0 / *na* | 0 / *na* | 18 / *na* | 31 / *na* | 36 current anxiety nos | 24 / *na* | 2 / *na* | 10 / *na* | *na* | *na* | | 15 | *n* = 1 SSRI/SNRI *n* = 4 antipsychotics *n* = 10 other medication nos |
| Cohort 3 / 4 | Structured Clinical Interview for DSM-IV TR | 95 (51) | 0 / 5 | 0 / 1 | 1 / 0 | 3 / 0 | 0 / 1 | 1 lifetime separation anxiety | 1 / 5 | 0 / 0 | 0 / 0 | 1 / 5 | 1 current ADHD, 1 current ODD, 7 lifetime ADHD, 1 lifetime ADD, 1 lifetime ODD, 1 lifetime Tourettes | | *na* | *na* |
| Generation R - sample with behavioral observations | Diagnostic Interview Schedule for Young Children (DISC-YC) (note: at age 5 – 8) | 584 (297) | 4 | *na* | *na* | 2 | 10 | 0 | 0 | *0* | 0 | *na* | *na* | *na* | | *na* |
| Generation R - sample with questionnaire data | Diagnostic Interview Schedule for Young Children (DISC-YC) (note: at age 5 – 8) | 1982 (1030 | 12 | *na* | *na* | 5 | 37 | 7 | 3 | *2* | 0 | *na* | *na* | *na* | | *na* |
| Maryland-PAX | Structured Clinical Interview for DSM-5 (SCID-5-RV) | 220 (109) | 0 / 20 | 0 / 1 | 0 / 0 | 0 / 0 | 3 / 17 | 30 / 21 | 0 / 35 | *na* | 0 / 3 | 14 / 2 | 14 / 45 | 0 | | *na* |
| Maryland-TAX | Mini-International Neuropsychiatric Interview For Children And Adolescents (MINI-KID; Sheehan et al., 1998) based on DSM-IV and ICD-10 criteria | 53 (28) | 24 / *na* | 4 / *na* | *na* | 12 / *na* | 4 / *na* | *na* | 8 / *na* | *na* | 3 / *na* | *na* | 2 current ADHD | 0 | | *na* |

|  |  |  | **Psychopathology based on clinical interview (current / lifetime but not current)** | | | | | | | | | | | |  | |
| --- | --- | --- | --- | --- | --- | --- | --- | --- | --- | --- | --- | --- | --- | --- | --- | --- |
|  |  |  | **Anxiety disorders** | | | | | | **Other psychopathology** | | | | | | **Psychotropic medication at time scan** | |
| **Sample** | **Clinical interview** | ***n* (*n* female)** | **SAD** | **PD** | **AG** | **GAD** | **SPH** | **Other anxiety disorders** | **MDD** | **OCD** | **PTSD** | **SUD** | **Other** | | ***n*** | **Specification medication** |
| Nijmegen Longitudinal Study | *na* | 71 (31) | *na* | *na* | *na* | *na* | *na* | *na* | *na* | *na* | *na* | *na* | *na* | *na* | | *na* |
| Pittsburgh | K-SADS-PL administered to parent and child | 15 (3) | 0 /1 | 0 / 1 | 0 / 0 | 0 / 0 | 0 / 1 | *n* = 4 simple phobia (lifetime); *n*  = 1 overanxious disorder (lifetime) | 0 / 1 | 0 / 1 | 0 / 0 | 1 / 3 | *na* | 0 | | *na* |
| San Raffaele | K-SADS-PL adminstered to parents | 20 (8) | 5 / *na* | 0 / *na* | 0 | 2 / *na* | *na* | *na* | 0 | 0 | 0 | *na* | *na* | 0 | | *na* |
| SDAN | K-SADS-PL administered to parent and child | 55 (26) | 18 / 2 | 0 / 0 | 0 / 0 | 24 / 4 | 8 / 4 | *n*  = 16 current and  *n*  = 7 lifetime separation anxiety disorder | 0 / 0 | 2 / 2 | 0 / 0 | 0 / 0 | *n*  = 6 current,  *n*  =1 lifetime ADHD / ODD | *na* | | *na* |
| Stony Brook Temperament Study | K-SADS-PL administered to parent and child | 74 (31) | 1 / 2 | 0 / 0 | 0 / 0 | 0 / 4 | 1 / 7 | *n*  = 3 lifetime (not current) separation anxiety | 0 / 1 | 0 / 1 | 0 / 0 | 0 / 0 | *n*  = 10 other psychopathology (ADHD, ODD, DBD) | 1 | | ADHD medication |
| TOTS | K-SADS-PL administered to parent and child | 96 (56) | 4 / 2 | 0 / 0 | 0 / 0 | 3 / 1 | 3 / 1 | *n =*  5 lifetime (not current) separation anxiety; *n*  = 1 current separation anxiety; *n*  = 1 current anxiety disorder nos | 1 / 3 | 0 / 0 | 1 / 0 | 0 / 0 | *n*  = 9 lifetime ADHD/ADD; *n*  = 7 current ADHD | 1 | | ADHD medication |
| Vanderbilt - children | K-SADS-PL | 55 (33) | 4 / 0 | 0 /0 | 0 / 0 | 0 /0 | 1 / 2 | *na* | 0 / 0 | 0 / 0 | 0 / 0 | 0 / 0 | *na* | 0 | | *na* |
| Vanderbilt - young adults | Structured Clinical Interview for DSM-IV | 150 (83) | 15 / 1 | 2 / 0 | 1 / 0 | 3 / 0 | 9 / 1 | *n* = 3 anxiety nos | 5 / 0 | 1 /0 | 1 / 0 | 3 / 2 | *n* = 2 dysthymia (current) | 0 | | *na* |
| Western University | K-SADS-PL administered to parent and child | 87 (38) | 1/ 1 | 0 / 0 | 0 / 0 | 1 / 1 | 1 / 0 | *na* | 0 / 0 | *na* | 0 / 0 | *na* | *n* = 5 current*,*  *n* = 1 lifetime (ADHD, ODD) | *na* | | *na* |

|  |  |  | **Psychopathology based on clinical interview (current / lifetime but not current)** | | | | | | | | | | | |  | |
| --- | --- | --- | --- | --- | --- | --- | --- | --- | --- | --- | --- | --- | --- | --- | --- | --- |
|  |  |  | **Anxiety disorders** | | | | | | **Other psychopathology** | | | | | | **Psychotropic medication at time scan** | |
| **Sample** | **Clinical interview** | ***n* (*n* female)** | **SAD** | **PD** | **AG** | **GAD** | **SPH** | **Other anxiety disorders** | **MDD** | **OCD** | **PTSD** | **SUD** | **Other** | | ***n*** | **Specification medication** |
| Wisconsin Twin Project - RDoC twin study | *na* | 316 (145) | *na* | *na* | *na* | *na* | *na* | *na* | *na* | *na* | *na* | *na* | *na* | *na* | | *na* |

**Supplemental Table 3b Clinical characteristics per sample – symptomatology at (around) time of scan**

|  | **Questionnaires – scores represent mean ± SD** | | | | | | | | | | | | | | | | | | |  |
| --- | --- | --- | --- | --- | --- | --- | --- | --- | --- | --- | --- | --- | --- | --- | --- | --- | --- | --- | --- | --- |
| **Sample** | **STAI-trait** | **ASI** | **BAI** | **LSAS - total** | **PSWQ** | **BDI** | **CDI** | **SCARED-T-P** | **SCARED-T-C** | **SCARED-SC-P** | **SCARED-SC-C** | **SCARED-PN-P** | **SCARED-PN-C** | **SCARED-GD-P** | **SCARED-GD-C** | **SCARED-SH-P** | **SCARED-SH-C** | **YSR - total** | **YSR - internalizing** | **Note** |
| Brains study | *na* | *na* | *na* | *na* | *na* | *na* | *na* | 11.3 ± 9.1 | 17.2 ± 11.8 | 4.0 ± 3.5 | 4.8 ± 3.6 | 0.9 ± 1.4 | 3.1 ± 3.5 | 3.8 ± 3.5 | 3.8 ± 3.5 | 0.4 ± 0.7 | 1.0 ± 1.2 | *na* | *na* | Data availability for SCARED subscales:  *n*  = 104 (child-report) and *n=* 118 (parent-report) |
| Brazilian High Risk Cohort | *na* | *na* | *na* | *na* | *na* | *na* | *na* | *na* | 24.6 ± 15.0 | *na* | 5.7 ± 3.5 | *na* | 4.7 ± 5.1 | *na* | 6.0 ± 4.5 | *na* | 1.5 ± 1.7 | *na* | *na* |  |
| Cohort 3 / 4 | *na* | *na* | *na* | *na* | *na* | *na* | *na* | 6.9 ± 6.5 | 12.3 ± 9.7 | 2.5 ± 2.7 | 3.5 ± 3.0 | 0.9 ± 1.5 | 2.4 ± 2.3 | 2.8 ± 2.9 | 4.2 ± 3.8 | 0.5 ± 0.9 | 0.9 ± 1.1 | *na* | *na* | Data availability for diagnostic information:  *n*  = 69 participants; data availability for SCARED: range 40 - 43 participants. |
| Generation R - sample with behavioral observations | *na* | *na* | *na* | *na* | *na* | *na* | *na* | *na* | *na* | *na* | *na* | *na* | *na* | *na* | *na* | *na* | *na* | *na* | *na* |  |
| Generation R - sample with questionnaire data | \| *na* \| *na* \| *na* \| *na* \| *na* \| *na* \| *na* \| \| --- \| --- \| --- \| --- \| --- \| --- \| --- \| | *na* | *na* | *na* | *na* | *na* | *na* | *na* | *na* | *na* | *na* | *na* | *na* | *na* | *na* | *na* | *na* | *na* | *na* |  |
| Maryland-PAX | *na* | *na* | *na* | 42.2 ± 22.3 | *na* | *na* | *na* | *na* | *na* | *na* | *na* | *na* | *na* | *na* | *na* | *na* | *na* | *na* | *na* |  |
| Maryland-TAX | 45.5 ± 13.7 | *na* | *na* | *na* | 48.2 ± 14.6 | *na* | *na* | 17.1 ± 14.5 | 27.9 ± 20.3 | 6.0 ± 4.4 | 7.1 ± 4.9 | 2.9 ± 4.1 | 6.4 ± 6.8 | 5.2 ± 4.8 | 8.7 ± 5.5 | 1.3 ± 1.8 | 2.2 ± 2.3 | *na* | *na* | Data on SCARED for 48 participants. |
| Nijmegen Longitudinal Study | *na* | *na* | *na* | *na* | *na* | *na* | *na* | *na* | *na* | *na* | *na* | *na* | *na* | *na* | *na* | *na* | *na* | *na* | *na* |  |
| Pittsburgh | *na* | *na* | *na* | *na* | *na* | *na* | *na* | *na* | *na* | *na* | *na* | *na* | *na* | *na* | *na* | *na* | *na* | *na* | *na* |  |
| San Raffaele | *na* | *na* | *na* | 27.6 ± 21.3 | *na* | *na* | *na* | *na* | *na* | 3.9 ± 3.2 | 3.9 ± 2.9 | *na* | *na* | *na* | *na* | *na* | *na* | *na* | *na* |  |
| SDAN | 34.9 ± 8.3 | *na* | *na* | *na* | 17.5 ± 9.9 | *na* | *na* | 21.5 ± 15.1 | 21.8 ± 15.1 | 5.6 ± 4.9 | 5.3 ± 3.8 | 2.6 ± 3.0 | 3.7 ± 4.2 | 7.1 ± 5.1 | 6.1 ± 4.5 | 1.6 ± 1.8 | 1.6 ± 1.7 | *na* | *na* | Data on PSWQ for 49 participants |
| Stony Brook Temperament Study | *na* | *na* | *na* | *na* | *na* | *na* | 3.8 ± 3.7 | 8.6 ± 7.4 | *na* | 2.7 ± 2.9 | *na* | 3.0 ± 0.7 | *na* | 3.0 ± 3.0 | *na* | 0.6 ± 1.0 | *na* | *na* | *na* | CDI: rated by child |

|  | **Questionnaires – scores represent mean ± SD** | | | | | | | | | | | | | | | | | | |  |
| --- | --- | --- | --- | --- | --- | --- | --- | --- | --- | --- | --- | --- | --- | --- | --- | --- | --- | --- | --- | --- |
| **Sample** | **STAI-trait** | **ASI** | **BAI** | **LSAS - total** | **PSWQ** | **BDI** | **CDI** | **SCARED-T-P** | **SCARED-T-C** | **SCARED-SC-P** | **SCARED-SC-C** | **SCARED-PN-P** | **SCARED-PN-C** | **SCARED-GD-P** | **SCARED-GD-C** | **SCARED-SH-P** | **SCARED-SH-C** | **YSR - total** | **YSR - internalizing** | **Note** |
| TOTS | 28.0 ± 6.3 | *na* | *na* | *na* | *na* | *na* | *na* | 9.6 ± 7.7 | 17.1 ± 9.7 | 3.5 ± 3.6 | 5.5 ± 3.4 | 1.7 ± 1.8 | 2.7 ± 2.6 | 2.8 ± 2.8 | 4.3 ± 3.5 | 0.6 ± 0.9 | 1.3 ± 1.2 | *na* | *na* | Data availability for psychopathology: *n* = 93; for SCARED: 35 - 43 participants. |
| Vanderbilt - children | *na* | *na* | *na* | *na* | *na* | *na* | 5.3 ± 5.5 | 11.5 ± 10.6 | 17.7 ± 13.5 | 4.4 ± 4.5 | 5.0 ± 3.6 | 1.0 ± 1.8 | 2.8 ± 3.3 | 3.2 ± 3.1 | 4.0 ± 3.4 | 0.3 ± 0.6 | 1.3 ± 1.5 | *na* | *na* | Data availability for questionnaires varies from  *n* = 44 to *n* = 47 |
| Vanderbilt - young adults | 31.8 ± 10.9 | 13.5 ± 10.9 | 6.4 ± 5.4 | 34.8 ± 21.7 | 46.6 ± 16.5 | 5.5 ± 6.6 | *na* | *na* | *na* | *na* | *na* | *na* | *na* | *na* | *na* | *na* | *na* | *na* | *na* | Data availability for questionnaires varies from  *n* = 29 *to n* = 149 |
| Western University | *na* | *na* | *na* | *na* | *na* | *na* | 5. 0 ± 5.3 | *na* | *na* | *na* | *na* | *na* | *na* | *na* | *na* | *na* | *na* | 36.1 ± 24.0 | 13.1 ± 9.6 | Data availability for psychopathology: *n = 82* |
| Wisconsin Twin Project - RDoC twin study | *Not yet available* | *na* | *na* | *na* | *Not yet available* | *Not yet available* | *na* | *na* | *na* | *na* | *na* | *na* | *na* | *na* | *na* | *na* | *na* | *na* | *na* |  |

**Supplemental Table 4 Overview of included independent variables per sample**

*Will be included in final submission (i.e. after performing the analyses)*

**References Supplemental Materials**

1. Bas-Hoogendam JM, Groenewold NA, Aghajani M, et al. ENIGMA-anxiety working group: Rationale for and organization of large-scale neuroimaging studies of anxiety disorders. *Hum Brain Mapp*. 2020:1-30. doi:10.1002/hbm.25100

2. Hamilton M. The assessment of anxiety states by rating. *Br J Med Psychol*. 1959;32(1):50-55. doi:10.1111/j.2044-8341.1959.tb00467.x

3. Meyer TJ, Miller ML, Metzger RL, Borkovec TD. Development and validation of the penn state worry questionnaire. *Behav Res Ther*. 1990;28(6):487-495.

4. Spitzer RL, Kroenke K, Williams JBW, Löwe B. A Brief Measure for Assessing Generalized Anxiety Disorder: The GAD-7. *Arch Intern Med*. 2006;166(10):1092-1097. doi:10.1001/archinte.166.10.1092

5. Spielberger CD, Gorsuch RL, Lushene RE. *STAI Manual for the State-Trait Anxiety Inventory*. Palo Alto, CA: Consulting Psychologists Press; 1970.

6. Taylor S, Zvolensky MJ, Cox BJ, et al. Robust dimensions of anxiety sensitivity: development and initial validation of the Anxiety Sensitivity Index-3. *Psychol Assess*. 2007;19(2):176.

7. Beck AT, Steer RA, Carbin MG. Psychometric properties of the Beck Depression Inventory: Twenty-five years of evaluation. *Clin Psychol Rev*. 1988;8(1):77-100. doi:10.1016/0272-7358(88)90050-5

8. Heimberg RG, Horner KJ, Juster HR, et al. Psychometric properties of the Liebowitz Social Anxiety Scale. *Psychol Med*. 1999;29(01):199-212. doi:10.1017/S0033291798007879

9. Bandelow B. Assessing the efficacy of treatments for panic disorder and agoraphobia: II. The Panic and Agoraphobia Scale. *Int Clin Psychopharmacol*. 1995;10(2):73-81. doi:10.1097/00004850-199506000-00003

10. Chambless DL, Caputo GC, Bright P, Gallagher R. Assessment of fear of fear in agoraphobics: The Body Sensations Questionnaire and the Agoraphobic Cognitions Questionnaire. *J Consult Clin Psychol*. 1984;52(6):1090-1097. doi:10.1037/0022-006X.52.6.1090

11. Shear MK, Rucci P, Williams J, et al. Reliability and validity of the Panic Disorder Severity Scale: replication and extension. *J Psychiatr Res*. 2001;35(5):293-296.

12. Birmaher B, Brent DA, Chiapetta L, Bridge J, Monga S, Baugher M. Psychometric Properties of the Screen for Child Anxiety Related Emotional Disorders (SCARED): A Replication Study. *J Am Acad Child Adolesc Psychiatry*. 1999;38(10):1230-1236. doi:https://doi.org/10.1097/00004583-199910000-00011

13. Beck AT, Steer R, Brown G. *Manual for the Beck Depression Inventory-II.* San Antonio, TX: Psychological Corporation; 1996.

14. Kovacs M. The Children’s Depression Inventory (CDI). *Psychopharmacol Bull*. 1985;21:995-998.

15. Hamilton M. A rating scale for depression. *J Neurol Neurosurg Psychiatry*. 1960;23(1):56-62. doi:10.1136/jnnp.23.1.56

16. Fu X, Taber-Thomas BC, Pérez-Edgar K. Frontolimbic functioning during threat-related attention: Relations to early behavioral inhibition and anxiety in children. *Biol Psychol*. 2017;122:98-109. doi:https://doi.org/10.1016/j.biopsycho.2015.08.010

17. Morales S, Taber-Thomas BC, Pérez-Edgar KE. Patterns of attention to threat across tasks in behaviorally inhibited children at risk for anxiety. *Dev Sci*. January 2017. doi:10.1111/desc.12391

18. Taber-Thomas BC, Morales S, Hillary FG, Pérez-Edgar KE. Altered topography of intrinsic functional connectivity in childhood risk for social anxiety. *Depress Anxiety*. 2016;33:995-1004. doi:10.1002/da.22508

19. Liu P, Taber-Thomas BC, Fu X, Pérez-Edgar KE. Biobehavioral Markers of Attention Bias Modification in Temperamental Risk for Anxiety: A Randomized Control Trial. *J Am Acad Child Adolesc Psychiatry*. 2018;57(2):103-110. doi:https://doi.org/10.1016/j.jaac.2017.11.016

20. Auday ES, Pérez-Edgar KE. Limbic and prefrontal neural volume modulate social anxiety in children at temperamental risk. *Depress Anxiety*. 2019;36(8):690-700. doi:10.1002/da.22941

21. Thai N, Taber-Thomas BC, Pérez-Edgar KE. Neural correlates of attention biases, behavioral inhibition, and social anxiety in children: An ERP study. *Dev Cogn Neurosci*. 2016;19:200-210. doi:https://doi.org/10.1016/j.dcn.2016.03.008

22. Suarez GL, Morales S, Metcalf K, Pérez-Edgar KE. Perinatal complications are associated with social anxiety: Indirect effects through temperament. *Infant Child Dev*. 2019;28(3):e2130. doi:https://doi.org/10.1002/icd.2130

23. Anaya B, Vallorani AM, Pérez-Edgar K. Individual dynamics of delta–beta coupling: using a multilevel framework to examine inter- and intraindividual differences in relation to social anxiety and behavioral inhibition. *J Child Psychol Psychiatry*. 2021;62(6):771-779. doi:https://doi.org/10.1111/jcpp.13319

24. Anaya B, Vallorani A, Pérez-Edgar K. Dyadic behavioral synchrony between behaviorally inhibited and non-inhibited peers is associated with concordance in EEG frontal Alpha asymmetry and Delta-Beta coupling. *Biol Psychol*. 2021;159:108018. doi:https://doi.org/10.1016/j.biopsycho.2021.108018

25. Wise S, Huang-Pollock C, Pérez-Edgar K. Implementation of the diffusion model on dot-probe task performance in children with behavioral inhibition. *Psychol Res*. 2021. doi:10.1007/s00426-021-01532-3

26. Bishop G, Spence SH, Mcdonald C. Can Parents and Teachers Provide a Reliable and Valid Report of Behavioral Inhibition? *Child Dev*. 2003;74(6):1899-1917. doi:https://doi.org/10.1046/j.1467-8624.2003.00645.x

27. Broeren S, Muris P. A Psychometric Evaluation of the Behavioral Inhibition Questionnaire in a Non-Clinical Sample of Dutch Children and Adolescents. *Child Psychiatry Hum Dev*. 2010;41(2):214-229. doi:10.1007/s10578-009-0162-9

28. Shaffer D, Fisher P, Lucas CP, Dulcan MK, Schwab-Stone ME. NIMH Diagnostic Interview Schedule for Children Version IV (NIMH DISC-IV): description, differences from previous versions, and reliability of some common diagnoses. *J Am Acad Child Adolesc Psychiatry*. 2000;39(1):28-38.

29. Hale WW, Raaijmakers Q, Muris P, Meeus W. Psychometric Properties of the Screen for Child Anxiety Related Emotional Disorders (SCARED) in the General Adolescent Population. *J Am Acad Child Adolesc Psychiatry*. 2005;44(3):283-290. doi:https://doi.org/10.1097/00004583-200503000-00013

30. Muris P, Dreessen L, Bögels S, Weckx M, van Melick M. A questionnaire for screening a broad range of DSM-defined anxiety disorder symptoms in clinically referred children and adolescents. *J Child Psychol Psychiatry*. 2004;45(4):813-820. doi:https://doi.org/10.1111/j.1469-7610.2004.00274.x

31. Dyson MW, Klein DN, Olino TM, Dougherty LR, Durbin CE. Social and Non-Social Behavioral Inhibition in Preschool-Age Children: Differential Associations with Parent-Reports of Temperament and Anxiety. *Child Psychiatry Hum Dev*. 2011;42(4):390-405. doi:10.1007/s10578-011-0225-6

32. Salum GA, Gadelha A, Pan PM, et al. High risk cohort study for psychiatric disorders in childhood: rationale, design, methods and preliminary results. *Int J Methods Psychiatr Res*. 2015;24(1):58-73. doi:10.1002/mpr.1459

33. Moura LM, Crossley NA, Zugman A, et al. Coordinated brain development: exploring the synchrony between changes in grey and white matter during childhood maturation. *Brain Imaging Behav*. 2017;11(3):808-817. doi:10.1007/s11682-016-9555-0

34. Axelrud LK, Simioni AR, Pine DS, et al. Neuroimaging Association Scores: reliability and validity of aggregate measures of brain structural features linked to mental disorders in youth. *Eur Child Adolesc Psychiatry*. 2020. doi:10.1007/s00787-020-01653-x

35. Hoffmann MS, Pan PM, Manfro GG, et al. Cross-Sectional and Longitudinal Associations of Temperament and Mental Disorders in Youth. *Child Psychiatry Hum Dev*. 2019;50(3):374-383. doi:10.1007/s10578-018-0846-0

36. Fleitlich-Bilyk B, Goodman R. Prevalence of child and adolescent psychiatric disorders in southeast Brazil. *J Am Acad Child Adolesc Psychiatry*. 2004;43(6):727-734.

37. Goodman R, Ford T, Richards H, Gatward R, Meltzer H. The Development and Well-Being Assessment: Description and Initial Validation of an Integrated Assessment of Child and Adolescent Psychopathology. *J Child Psychol Psychiatry*. 2000;41(5):645-655. doi:10.1111/j.1469-7610.2000.tb02345.x

38. Kamakura W, Mazzon JA. Socioeconomic stratification criteria and classification tools in Brazil/Criterios de estratificacao e comparacao de classificadores socioeconomicos no Brasil/Criterios de estratificación y comparación de clasificadores socioeconómicos en Brasil. *RAE*. 2016;56(1):55-71.

39. Ellis LK, Rothbart MK. Revision of the early adolescent temperament questionnaire. In: *Poster Presented at the 2001 Biennial Meeting of the Society for Research in Child Development, Minneapolis, Minnesota*. Citeseer; 2001.

40. Laceulle OM, Ormel J, Vollebergh WAM, Van Aken MAG, Nederhof E. A test of the vulnerability model: temperament and temperament change as predictors of future mental disorders–the TRAILS study. *J Child Psychol Psychiatry*. 2014;55(3):227-236.

41. Rothbart MK. Temperament, development, and personality. *Curr Dir Psychol Sci*. 2007;16(4):207-212.

42. Fox NA, Henderson HA, Rubin KH, Calkins SD, Schmidt LA. Continuity and discontinuity of behavioral inhibition and exuberance: Psychophysiological and behavioral influences across the first four years of life. *Child Dev*. 2001;72(1):1-21. doi:https://doi.org/10.1111/1467-8624.00262

43. Shechner T, Fox NA, Mash JA, et al. Differences in neural response to extinction recall in young adults with or without history of behavioral inhibition. *Dev Psychopathol*. 2018;30(1):179-189.

44. Roy AK, Benson BE, Degnan KA, et al. Alterations in amygdala functional connectivity reflect early temperament. *Biol Psychol*. 2014;103:248-254. doi:10.1016/j.biopsycho.2014.09.007

45. First MB, Spitzer RL. Structured Clinical Interview for DSM-IV-TR Axis I Disorders, Research Version, Non-patient Edition. *SCIDI/NP) New York Biometrics Res New York State Psychiatr Inst*. 2002.

46. Goldsmith HH, Reilly J, Lemery KS, Longley S, Prescott A. Laboratory temperament assessment battery: Preschool version. *Unpubl Manuscr*. 1995.

47. Goldsmith HH. Studying temperament via construction of the Toddler Behavior Assessment Questionnaire. *Child Dev*. 1996;67(1):218-235.

48. Jaddoe VW V, van Duijn CM, van der Heijden AJ, et al. The Generation R Study: design and cohort update until the age of 4 years. *Eur J Epidemiol*. 2008;23(12):801. doi:10.1007/s10654-008-9309-4

49. White T, Marroun H El, Nijs I, et al. Pediatric population-based neuroimaging and the Generation R Study: the intersection of developmental neuroscience and epidemiology. *Eur J Epidemiol*. 2013;28(1):99-111. doi:10.1007/s10654-013-9768-0

50. White T, Muetzel RL, El Marroun H, et al. Paediatric population neuroimaging and the Generation R Study: the second wave. *Eur J Epidemiol*. 2018;33(1):99-125. doi:10.1007/s10654-017-0319-y

51. Pappa I, Mileva-Seitz VR, Szekely E, et al. DRD4 VNTRs, observed stranger fear in preschoolers and later ADHD symptoms. *Psychiatry Res*. 2014;220(3):982-986. doi:https://doi.org/10.1016/j.psychres.2014.09.004

52. Jansen PW, Raat H, Mackenbach JP, et al. Socioeconomic inequalities in infant temperament. *Soc Psychiatry Psychiatr Epidemiol*. 2009;44(2):87-95. doi:10.1007/s00127-008-0416-z

53. Jaddoe VW V, Mackenbach JP, Moll HA, et al. The Generation R Study: design and cohort profile. *Eur J Epidemiol*. 2006;21(6):475-484.

54. Fisher P, Lucas C. Diagnostic interview schedule for children (DISC-IV)-young child. *New York Columbia Univ*. 2006.

55. Gartstein MA, Rothbart MK. Studying infant temperament via the revised infant behavior questionnaire. *Infant Behav Dev*. 2003;26(1):64-86.

56. Roza SJ, Van Lier PAC, Jaddoe VW V, et al. Intrauterine growth and infant temperamental difficulties: the Generation R Study. *J Am Acad Child Adolesc Psychiatry*. 2008;47(3):264-272.

57. Goldsmith HH, Reilly J, Lemery KS, Longley S, Prescott A. The laboratory assessment battery: Preschool version (LAB-TAB). *Madison Univ Wisconsin*. 1999.

58. Hur J, Smith JF, DeYoung KA, et al. Anxiety and the Neurobiology of Temporally Uncertain Threat Anticipation. *J Neurosci*. 2020;40(41):7949 LP - 7964. doi:10.1523/JNEUROSCI.0704-20.2020

59. Hur J, DeYoung KA, Islam S, Anderson AS, Barstead MG, Shackman AJ. Social context and the real-world consequences of social anxiety. *Psychol Med*. 2020;50(12):1989-2000. doi:DOI: 10.1017/S0033291719002022

60. Hur J, Kuhn M, Grogans SE, et al. Anxiety-related frontocortical activity is associated with dampened stressor reactivity in the real world. *Psychol Sci*. 2022;in press. doi:10.1101/2021.03.17.435791

61. First MB, Williams JB, Karg RS, Spitzer R. *Structured Clinical Interview for DSM-5, Research Version (SCID-5 for DSM-5, Research Version; SCID-5-RV).* Arlington, VA: American Psychiatric Association; 2015.

62. Gladstone G, Parker G. Measuring a behaviorally inhibited temperament style: Development and initial validation of new self-report measures. *Psychiatry Res*. 2005;135(2):133-143. doi:https://doi.org/10.1016/j.psychres.2005.03.005

63. Bunnell BE, Beidel DC, Liu L, Joseph DL, Higa-McMillan C. The SPAIC-11 and SPAICP-11: Two brief child-and parent-rated measures of social anxiety. *J Anxiety Disord*. 2015;36:103-109.

64. Sheehan D V., Lecrubier Y, Sheehan KH, et al. The Mini-International Neuropsychiatric Interview (M.I.N.I.): the development and validation of a structured diagnostic psychiatric interview for DSM-IV and ICD-10. *J Clin Psychiatry*. 1998;59 Suppl 2:22-33.

65. Reznick JS, Hegeman IM, Kaufman ER, Woods SW, Jacobs M. Retrospective and concurrent self-report of behavioral inhibition and their relation to adult mental health. *Dev Psychopathol*. 1992;4(2):301-321. doi:DOI: 10.1017/S095457940000016X

66. Tyborowska A, Volman I, Niermann HCM, et al. Early-life and pubertal stress differentially modulate grey matter development in human adolescents. *Sci Rep*. 2018;8(1):9201. doi:10.1038/s41598-018-27439-5

67. Van Bakel HJA, Riksen‐Walraven JM. Stress reactivity in 15‐month‐old infants: Links with infant temperament, cognitive competence, and attachment security. *Dev Psychobiol J Int Soc Dev Psychobiol*. 2004;44(3):157-167.

68. Van Bakel HJA, Riksen‐Walraven JM. Parenting and development of one‐year‐olds: Links with parental, contextual, and child characteristics. *Child Dev*. 2002;73(1):256-273.

69. Meyers SA. Mothering in context: Ecological determinants of parent behavior. *Merrill-Palmer Q*. 1999:332-357.

70. Achenbach TM, Ruffle TM. The Child Behavior Checklist and related forms for assessing behavioral/emotional problems and competencies. *Pediatr Rev*. 2000;21(8):265-271. doi:10.1542/pir.21-8-265

71. Goldsmith HH. The toddler behavior assessment questionnaire. *Oregon Cent Study Emot Tech Rep*. 1988;(88-04).

72. Mullen M, Snidman N, Kagan J. Brief report free-play behavior in inhibited and uninhibited children. *Infant Behav Dev*. 1993;16(3):383-389.

73. Nachmias M, Gunnar M, Mangelsdorf S, Parritz RH, Buss K. Behavioral inhibition and stress reactivity: The moderating role of attachment security. *Child Dev*. 1996;67(2):508-522.

74. Hill SY, Shen S, Lowers L, Locke-Wellman J, Matthews AG, McDermott M. Psychopathology in offspring from multiplex alcohol dependence families with and without parental alcohol dependence: a prospective study during childhood and adolescence. *Psychiatry Res*. 2008;160(2):155-166.

75. Hill SY, Tessner KD, McDermott MD. Psychopathology in offspring from families of alcohol dependent female probands: a prospective study. *J Psychiatr Res*. 2011;45(3):285-294.

76. Hill SY. Familial risk for alcohol dependence and brain morphology: the role of cortical thickness across the lifespan. *Alcohol Clin Exp Res*. 2018;42(5):841-844.

77. Hill SY, Wang S, Carter H, McDermott MD, Zezza N, Stiffler S. Amygdala volume in offspring from multiplex for alcohol dependence families: the moderating influence of childhood environment and 5-HTTLPR variation. *J Alcohol drug Depend*. 2013.

78. Robins LN, Helzer JE, Croughan J, Ratcliff KS. National Institute of Mental Health diagnostic interview schedule: Its history, characteristics, and validity. *Arch Gen Psychiatry*. 1981;38(4):381-389.

79. Chambers WJ, Puig-Antich J, Hirsch M, et al. The assessment of affective disorders in children and adolescents by semistructured interview: test-retest reliability of the Schedule for Affective Disorders and Schizophrenia for School-Age Children, Present Episode Version. *Arch Gen Psychiatry*. 1985;42(7):696-702.

80. Janča A, Robins LN, Cottler LB, Early TS. Clinical observation of assessment using the Composite International Diagnostic Interview (CIDI): an analysis of the CIDI field trials–wave II at the St Louis site. *Br J Psychiatry*. 1992;160(6):815-818.

81. Cottler LB, Robins LN, Helzer JE. The reliability of the CIDI‐SAM: a comprehensive substance abuse interview. *Br J Addict*. 1989;84(7):801-814.

82. Hill SY, Tessner K, Wang S, Carter H, McDermott M. Temperament at 5 years of age predicts amygdala and orbitofrontal volume in the right hemisphere in adolescence. *Psychiatry Res*. 2010;182(1):14-21. doi:10.1016/j.pscychresns.2009.11.006

83. Hill SY, Lowers L, Locke J, Snidman N, Kagan J. Behavioral Inhibition in Children From Families at High Risk for Developing Alcoholism. *J Am Acad Child Adolesc Psychiatry*. 1999;38(4):410-417. doi:https://doi.org/10.1097/00004583-199904000-00013

84. Kagan J, Reznick JS, Snidman N, Gibbons J, Johnson MO. Childhood derivatives of inhibition and lack of inhibition to the unfamiliar. *Child Dev*. 1988:1580-1589.

85. Hill SY, Tessner K, Wang S, Carter H, McDermott M. Temperament at 5 years of age predicts amygdala and orbitofrontal volume in the right hemisphere in adolescence. *Psychiatry Res*. 2010;182(1):14-21. doi:10.1016/j.pscychresns.2009.11.006

86. Tellegen A, Lykken DT, Bouchard TJ, Wilcox KJ, Segal NL, Rich S. Personality similarity in twins reared apart and together. *J Pers Soc Psychol*. 1988;54(6):1031.

87. Battaglia M, Zanoni A, Taddei M, et al. Cerebral responses to emotional expressions and the development of social anxiety disorder: a preliminary longitudinal study. *Depress Anxiety*. 2012;29(1):54-61. doi:10.1002/da.20896

88. Battaglia M, Ogliari A, Zanoni A, et al. Children’s discrimination of expressions of emotions: Relationship with indices of social anxiety and shyness. *J Am Acad Child Adolesc Psychiatry*. 2004;43(3):358-365.

89. Battaglia M, Ogliari A, Zanoni A, et al. Influence of the serotonin transporter promoter gene and shyness on children’s cerebral responses to facial expressions. *Arch Gen Psychiatry*. 2005;62(1):85-94.

90. Taddei M, Tettamanti M, Zanoni A, Cappa S, Battaglia M. Brain white matter organisation in adolescence is related to childhood cerebral responses to facial expressions and harm avoidance. *Neuroimage*. 2012;61(4):1394-1401.

91. Kaufman J, Birmaher B, Brent D, et al. Schedule for affective disorders and schizophrenia for school-age children-present and lifetime version (K-SADS-PL): initial reliability and validity data. *J Am Acad Child Adolesc Psychiatry*. 1997;36(7):980-988.

92. Biederman J, Hirshfeld-Becker DR, Rosenbaum JF, et al. Further evidence of association between behavioral inhibition and social anxiety in children. *Am J Psychiatry*. 2001;158(10):1673-1679. http://www.ncbi.nlm.nih.gov/pubmed/11579001. Accessed November 5, 2013.

93. Hirshfeld-Becker DR, Biederman J. Rationale and Principles for Early Intervention with Young Children at Risk for Anxiety Disorders. *Clin Child Fam Psychol Rev*. 2002;5(3):161-172. doi:10.1023/A:1019687531040

94. DUMMIT III ES, Klein RG, Tancer NK, Asche B, Martin J, Fairbanks JA. Systematic assessment of 50 children with selective mutism. *J Am Acad Child Adolesc Psychiatry*. 1997;36(5):653-660.

95. Masia CL, Klein RG, Storch EA, Corda B. School-based behavioral treatment for social anxiety disorder in adolescents: Results of a pilot study. *J Am Acad Child Adolesc Psychiatry*. 2001;40(7):780-786.

96. Cooper PJ, Eke M. Childhood shyness and maternal social phobia: A community study. *Br J Psychiatry*. 1999;174(5):439-443. doi:DOI: 10.1192/bjp.174.5.439

97. Stevenson‐Hinde J, Glover A. Shy girls and boys: A new look. *J Child Psychol Psychiatry*. 1996;37(2):181-187.

98. Cloninger CR, Przybeck TR, Svrakic DM, Wetzel RD. The Temperament and Character Inventory (TCI): A guide to its development and use. 1994.

99. Marshall PJ, Stevenson‐Hinde J. Behavioral inhibition, heart period, and respiratory sinus arrhythmia in young children. *Dev Psychobiol J Int Soc Dev Psychobiol*. 1998;33(3):283-292.

100. Švrakić NM, Švrakić DM, Cloninger CR. A general quantitative theory of personality development: fundamentals of a self-organizing psychobiological complex. *Dev Psychopathol*. 1996;8(1):247-272.

101. Battaglia M, Przybeck TR, Bellodi L, Cloninger CR. Temperament dimensions explain the comorbidity of psychiatric disorders. *Compr Psychiatry*. 1996;37(4):292-298.

102. Linke JO, Abend R, Kircanski K, et al. Shared and Anxiety-Specific Pediatric Psychopathology Dimensions Manifest Distributed Neural Correlates. *Biol Psychiatry*. 2021;89(6):579-587. doi:https://doi.org/10.1016/j.biopsych.2020.10.018

103. Gold AL, Brotman MA, Adleman NE, et al. Comparing Brain Morphometry Across Multiple Childhood Psychiatric Disorders. *J Am Acad Child Adolesc Psychiatry*. 2016;55(12):1027-1037. doi:10.1016/j.jaac.2016.08.008

104. Gold AL, Abend R, Britton JC, et al. Age differences in the neural correlates of anxiety disorders: An fMRI study of response to learned threat. *Am J Psychiatry*. 2020;177(5):454-463.

105. Kim J, Klein DN, Olino TM, Dyson MW, Dougherty LR, Durbin CE. Psychometric Properties of the Behavioral Inhibition Questionnaire in Preschool Children. *J Pers Assess*. 2011;93(6):545-555. doi:10.1080/00223891.2011.608756

106. Laptook RS, Klein DN, Olino TM, Dyson MW, Carlson G. Low positive affectivity and behavioral inhibition in preschool-age children: A replication and extension of previous findings. *Pers Individ Dif*. 2010;48(5):547-551.

107. Johnson VC, Olino TM, Klein DN, et al. A longitudinal investigation of predictors of the association between age 3 and age 6 behavioural inhibition. *J Res Pers*. 2016;63:51-61. doi:https://doi.org/10.1016/j.jrp.2016.04.008

108. Stumper A, Danzig AP, Dyson MW, Olino TM, Carlson GA, Klein DN. Parents’ behavioral inhibition moderates association of preschoolers’ BI with risk for age 9 anxiety disorders. *J Affect Disord*. 2017;210:35-42. doi:https://doi.org/10.1016/j.jad.2016.12.008

109. Mumper EE, Dyson MW, Finsaas MC, Olino TM, Klein DN. Life stress moderates the effects of preschool behavioral inhibition on anxiety in early adolescence. *J Child Psychol Psychiatry*. 2020;61(2):167-174.

110. Kopala-Sibley DC, Cyr M, Finsaas MC, et al. Early Childhood Parenting Predicts Late Childhood Brain Functional Connectivity During Emotion Perception and Reward Processing. *Child Dev*. 2020;91(1):110-128. doi:https://doi.org/10.1111/cdev.13126

111. Kann SJ, O’Rawe JF, Huang AS, Klein DN, Leung H-C. Preschool negative emotionality predicts activity and connectivity of the fusiform face area and amygdala in later childhood. *Soc Cogn Affect Neurosci*. 2017;12(9):1511-1519.

112. Olino TM, Klein DN, Lewinsohn PM, Rohde P, Seeley JR. Latent trajectory classes of depressive and anxiety disorders from adolescence to adulthood: descriptions of classes and associations with risk factors. *Compr Psychiatry*. 2010;51(3):224-235. doi:https://doi.org/10.1016/j.comppsych.2009.07.002

113. Olino TM, Klein DN, Dyson MW, Rose SA, Durbin CE. Temperamental emotionality in preschool-aged children and depressive disorders in parents: associations in a large community sample. *J Abnorm Psychol*. 2010;119(3):468.

114. Egger HL, Ascher BH, Angold A. Preschool age psychiatric assessment (PAPA). *Durham (North Carolina) Duke Univ Med Cent*. 1999.

115. Durbin CE, Hayden EP, Klein DN, Olino TM. Stability of laboratory-assessed temperamental emotionality traits from ages 3 to 7. *Emotion*. 2007;7(2):388-399. doi:10.1037/1528-3542.7.2.388

116. Kagan J. Temperamental contributions to social behavior. *Am Psychol*. 1989;44(4):668.

117. Durbin CE, Klein DN, Hayden EP, Buckley ME, Moerk KC. Temperamental emotionality in preschoolers and parental mood disorders. *J Abnorm Psychol*. 2005;114(1):28.

118. Hane AA, Fox NA, Henderson HA, Marshall PJ. Behavioral reactivity and approach-withdrawal bias in infancy. *Dev Psychol*. 2008;44(5):1491.

119. Buzzell GA, Troller-Renfree S V., Barker T V., et al. A Neurobehavioral Mechanism Linking Behaviorally Inhibited Temperament and Later Adolescent Social Anxiety. *J Am Acad Child Adolesc Psychiatry*. 2017;56(12):1097-1105. doi:10.1016/j.jaac.2017.10.007

120. Filippi CA, Sachs JF, Phillips D, et al. Infant behavioral reactivity predicts change in amygdala volume 12 years later. *Dev Cogn Neurosci*. 2020;42:100776. doi:https://doi.org/10.1016/j.dcn.2020.100776

121. Calkins SD, Fox NA, Marshall TR. Behavioral and physiological antecedents of inhibited and uninhibited behavior. *Child Dev*. 1996;67(2):523-540.

122. Birmaher B, Khetarpal S, Brent D, et al. The screen for child anxiety related emotional disorders (SCARED): Scale construction and psychometric characteristics. *J Am Acad Child Adolesc Psychiatry*. 1997;36(4):545-553.

123. Goldsmith HH, Rothbart MK. The laboratory temperament assessment battery (Lab-TAB): Pre-locomotor Version 3.1. *Dep Psychol Univ Oregon*. 1999.

124. Clauss JA, Benningfield MM, Rao U, Blackford JU. Altered Prefrontal Cortex Function Marks Heightened Anxiety Risk in Children. *J Am Acad Child Adolesc Psychiatry*. 2016;55(9):809-816. doi:10.1016/j.jaac.2016.05.024

125. Nebert DW. Extreme discordant phenotype methodology: an intuitive approach to clinical pharmacogenetics. *Eur J Pharmacol*. 2000;410(2-3):107-120.

126. NL KASK. Kaufman Brief Intelligence Test. Manual. *K-BIT) Am Guid Serv Circ Pines, MN*. 1990.

127. Oldfield R. The assessment and analysis of handedness: The Edinburgh inventory. *Neuropsychologia*. 1971;9(1):97-113. doi:10.1016/0028-3932(71)90067-4

128. Smucker MR, Craighead WE, Craighead LW, Green BJ. Normative and reliability data for the Children’s Depression Inventory. *J Abnorm Child Psychol*. 1986;14(1):25-39.

129. Avery SN, Blackford JU. Slow to warm up: the role of habituation in social fear. *Soc Cogn Affect Neurosci*. 2016;11(11):1832-1840. doi:10.1093/scan/nsw095

130. Blackford JU, Avery SN, Shelton RC, Zald DH. Amygdala temporal dynamics: temperamental differences in the timing of amygdala response to familiar and novel faces. *BMC Neurosci*. 2009;10:145. doi:10.1186/1471-2202-10-145

131. Blackford JU, Avery SN, Cowan RL, Shelton RC, Zald DH. Sustained amygdala response to both novel and newly familiar faces characterizes inhibited temperament. *Soc Cogn Affect Neurosci*. 2011;6(5):621-629. doi:10.1093/scan/nsq073

132. Clauss JA, Avery SN, Vanderklok RM, et al. Neurocircuitry underlying risk and resilience to Social Anxiety Disorder. *Depress Anxiety*. 2014;31(10):822-833. doi:10.1002/da.22265

133. Thornton-Wells TA, Avery SN, Blackford JU. Using novel control groups to dissect the amygdala’s role in Williams syndrome. *Dev Cogn Neurosci*. 2011;1(3):295-304. doi:https://doi.org/10.1016/j.dcn.2011.03.003

134. Blackford JU, Allen AH, Cowan RL, Avery SN. Amygdala and hippocampus fail to habituate to faces in individuals with an inhibited temperament. *Soc Cogn Affect Neurosci*. 2013;8(2):143-150. doi:10.1093/scan/nsr078

135. Spitzer RL, Williams JBW, Gibbon M, First MB. The structured clinical interview for DSM-III-R (SCID): I: history, rationale, and description. *Arch Gen Psychiatry*. 1992;49(8):624-629.

136. Rohrbacher H, Hoyer J, Beesdo K, et al. Psychometric properties of the Retrospective Self Report of Inhibition (RSRI) in a representative German sample. *Int J Methods Psychiatr Res*. 2008;17(2):80-88.

137. Clauss JA, Seay AL, Vanderklok RM, et al. Structural and functional bases of inhibited temperament. *Soc Cogn Affect Neurosci*. 2014;9(12):2049-2058. doi:10.1093/scan/nsu019

138. Liu P, Kryski KR, Smith HJ, Joanisse MF, Hayden EP. Transactional relations between early child temperament, structured parenting, and child outcomes: A three-wave longitudinal study. *Dev Psychopathol*. 2020;32(3):923-933. doi:DOI: 10.1017/S0954579419000841

139. Liu P, Vandermeer MRJ, Joanisse MF, Barch DM, Dozois DJA, Hayden EP. Neural Activity During Self-referential Processing in Children at Risk for Depression. *Biol Psychiatry Cogn Neurosci Neuroimaging*. 2020;5(4):429-437. doi:https://doi.org/10.1016/j.bpsc.2019.12.012

140. Vandermeer MRJ, Sheikh HI, Singh SS, et al. The BDNF gene val66met polymorphism and behavioral inhibition in early childhood. *Soc Dev*. 2018;27(3):543-554. doi:10.1111/sode.12292

141. Mohamed Ali O, Vandermeer MRJ, Sheikh HI, Joanisse MF, Hayden EP. Girls’ internalizing symptoms and white matter tracts in Cortico-Limbic circuitry. *NeuroImage Clin*. 2019;21:101650. doi:https://doi.org/10.1016/j.nicl.2018.101650

142. Vandermeer MRJ, Liu P, Mohamed Ali O, et al. Orbitofrontal cortex grey matter volume is related to children’s depressive symptoms. *NeuroImage Clin*. 2020;28:102395. doi:https://doi.org/10.1016/j.nicl.2020.102395

143. Liu P, Vandemeer MRJ, Joanisse MF, Barch DM, Dozois DJA, Hayden EP. Depressogenic self-schemas are associated with smaller regional grey matter volume in never-depressed preadolescents. *NeuroImage Clin*. 2020;28:102422. doi:https://doi.org/10.1016/j.nicl.2020.102422

144. Liu P, Vandermeer MRJ, Mohamed Ali O, et al. Maternal Depression, Child Temperament, and Early-Life Stress Predict Never-Depressed Preadolescents’ Functional Connectivity During a Negative-Mood Induction. *Clin Psychol Sci*. June 2021:21677026211016420. doi:10.1177/21677026211016419

145. Dunn LM, Dunn LM, Bulheller S, Häcker H. *Peabody Picture Vocabulary Test*. American Guidance Service Circle Pines, MN; 1965.

146. Kovacs M. The Childrens’ Depression Inventory: A self-rated depression scale for school-aged youngsters. *Pittsburgh Univ Pittsburgh Sch Med*. 1983.

147. Achenbach TM, Rescorla L. *Manual for the ASEBA School-Age Forms & Profiles: An Integrated System of Multi-Informant Assessment*. Aseba Burlington, VT:; 2001.

148. Rankin Williams L, Degnan KA, Perez-Edgar KE, et al. Impact of Behavioral Inhibition and Parenting Style on Internalizing and Externalizing Problems from Early Childhood through Adolescence. *J Abnorm Child Psychol*. 2009;37(8):1063-1075. doi:10.1007/s10802-009-9331-3

149. Schmidt NL, Brooker RJ, Carroll IC, et al. Longitudinal Research at the Interface of Affective Neuroscience, Developmental Psychopathology, Health and Behavioral Genetics: Findings from the Wisconsin Twin Project. *Twin Res Hum Genet*. 2019;22(4):233-239. doi:DOI: 10.1017/thg.2019.55

150. Schmidt NL, Lemery-Chalfant K, Goldsmith HH. Wisconsin Twin Project Overview: Temperament and Affective Neuroscience. *Twin Res Hum Genet*. 2019;22(6):794-799. doi:DOI: 10.1017/thg.2019.108

151. Schmidt NL, Van Hulle CA, Brooker RJ, Meyer LR, Lemery-Chalfant K, Goldsmith HH. Wisconsin Twin Research: Early development, childhood psychopathology, autism, and sensory over-responsivity. *Twin Res Hum Genet*. 2013;16(1):376-384.

152. Goldsmith HH, Lemery-Chalfant K, Schmidt NL, Arneson CL, Schmidt CK. Longitudinal analyses of affect, temperament, and childhood psychopathology. *Twin Res Hum Genet*. 2007;10(1):118-126.

153. Lemery-Chalfant K, Goldsmith HH, Schmidt NL, Arneson CL, Van Hulle CA. Wisconsin Twin Panel: current directions and findings. *Twin Res Hum Genet*. 2006;9(6):1030-1037.

154. Van Hulle CA, Lemery KS, Goldsmith HH. Wisconsin twin panel. *Twin Res Hum Genet*. 2002;5(5):502-505.

155. Moore MM, Planalp EM, Van Hulle CA, Goldsmith H. Pediatric assessment of Research Domain Criteria positive and negative valence systems: Partial genetic mediation of links to problem behaviors. *J Abnorm Psychol (in Press*.

156. Goldsmith HH, Reilly J, Lemery KS, Longley S, Prescott A. Preliminary manual for the Preschool Laboratory Temperament Assessment Battery (Technical Report Version 1.0). *Madison, WI Dep Psychol Univ Wisconsin—Madison*. 1993.

157. Gagne JR, Van Hulle CA, Aksan N, Essex MJ, Goldsmith HH. Deriving childhood temperament measures from emotion-eliciting behavioral episodes: scale construction and initial validation. *Psychol Assess*. 2011;23(2):337.

158. Goldsmith HH, Lemery KS, Schmidt NL, Schmidt CK, Chow OK, Justen AA. Field administration manual for the Laboratory Temperament Assessment Battery: Middle childhood version. *Psychol Dep Univ Wisconsin–Madison[Google Sch*. 2010.

159. Armstrong JM, Goldstein LH. Manual for the MacArthur health and behavior questionnaire (HBQ 1.0). *MacArthur Found Res Netw Psychopathol Dev Univ Pittsburgh*. 2003.

160. Rothbart MK, Ahadi SA, Hershey KL, Fisher P. Investigations of temperament at three to seven years: The Children’s Behavior Questionnaire. *Child Dev*. 2001;72(5):1394-1408.
